# Dopamine and reward hypersensitivity in Parkinson’s disease with impulse control disorder

**DOI:** 10.1093/brain/awaa198

**Published:** 2020-08-06

**Authors:** Daniel S Drew, Kinan Muhammed, Fahd Baig, Mark Kelly, Youssuf Saleh, Nagaraja Sarangmat, David Okai, Michele Hu, Sanjay Manohar, Masud Husain

**Affiliations:** a1 Nuffield Department of Clinical Neurosciences, University of Oxford, Level 6, West Wing, John Radcliffe Hospital, Oxford, OX3 9DU, UK; a2 Department of Experimental Psychology, University of Oxford, Anna Watts Building, Radcliffe Observatory Quarter, Woodstock Road, Oxford, OX2 6GG, UK; a3 Oxford Parkinson’s Disease Centre, Department of Psychiatry, University of Oxford, Warneford Hospital, Oxford, OX3 7JX, UK; a4 Institute of Molecular and Clinical Sciences, St. George’s University London, Blackshaw Road, Tooting, London, SW17 0QT, UK; a5 Department of Neuropsychiatry, Maudsley Outpatients, Denmark Hill, Maudsley Hospital, London, SE5 8AZ, UK; a6 Department of Psychological Medicine, Institute of Psychiatry, Psychology and Neuroscience, De Crespigny Park, Camberwell, London, SE5 8AF, UK; a7 Wellcome Centre for Integrative Neuroimaging, University of Oxford, Level 6, West Wing, John Radcliffe Hospital, Oxford, OX3 9DU, UK

**Keywords:** Parkinson’s disease, impulse control disorder, dopamine, pupillometry, reward sensitivity

## Abstract

Impulse control disorders in Parkinson’s disease are common neuropsychiatric complications associated with dopamine replacement therapy. Some patients treated with dopamine agonists develop pathological behaviours, such as gambling, compulsive eating, shopping, or disinhibited sexual behaviours, which can have a severe impact on their lives and that of their families. In this study we investigated whether hypersensitivity to reward might contribute to these pathological behaviours and how this is influenced by dopaminergic medication. We asked participants to shift their gaze to a visual target as quickly as possible, in order to obtain reward. Critically, the reward incentive on offer varied over trials. Motivational effects were indexed by pupillometry and saccadic velocity, and patients were tested ON and OFF dopaminergic medication, allowing us to measure the effect of dopaminergic medication changes on reward sensitivity. Twenty-three Parkinson’s disease patients with a history of impulse control disorders were compared to 26 patients without such behaviours, and 31 elderly healthy controls. Intriguingly, behavioural apathy was reported alongside impulsivity in the majority of patients with impulse control disorders. Individuals with impulse control disorders also exhibited heightened sensitivity to exogenous monetary rewards cues both ON and OFF (overnight withdrawal) dopamine medication, as indexed by pupillary dilation in anticipation of reward. Being OFF dopaminergic medication overnight did not modulate pupillary reward sensitivity in impulse control disorder patients, whereas in control patients reward sensitivity was significantly reduced when OFF dopamine. These effects were independent of cognitive impairment or total levodopa equivalent dose. Although dopamine agonist dose did modulate pupillary responses to reward, the pattern of results was replicated even when patients with impulse control disorders on dopamine agonists were excluded from the analysis. The findings suggest that hypersensitivity to rewards might be a contributing factor to the development of impulse control disorders in Parkinson’s disease. However, there was no difference in reward sensitivity between patient groups when ON dopamine medication, suggesting that impulse control disorders may not emerge simply because of a direct effect of dopaminergic drug level on reward sensitivity. The pupillary reward sensitivity measure described here provides a means to differentiate, using a physiological measure, Parkinson’s disease patients with impulse control disorder from those who do not experience such symptoms. Moreover, follow-up of control patients indicated that increased pupillary modulation by reward can be predictive of the risk of future emergence of impulse control disorders and may thereby provide the potential for early identification of patients who are more likely to develop these symptoms.

## Teaser

Impulse control disorders are common in Parkinson’s disease, but the underlying mechanisms and the reasons why only some patients are affected remain unclear. Drew *et al.* report that affected individuals are hypersensitive to rewards, and this hypersensitivity precedes and contributes to the development of impulsivity.

## Introduction

Idiopathic Parkinson’s disease is now known to be associated with a wide range of non-motor symptoms including potential impairment in cognition, mood and motivation (Chaudhuri *et al.*, 2011, 2006). Impulse control disorders (ICDs) are recognized to be important neuropsychiatric complications in Parkinson’s disease (Okai *et al.*, 2011; Averbeck *et al.*, 2014; Goerlich‐Dobre *et al.*, 2014; Ricciardi *et al.*, 2016; Voon *et al.*, 2017; Weintraub and Mamikonyan, 2019), with the most common ICDs being compulsive buying, gambling, eating and disinhibited sexual behaviour (Housden *et al.*, 2010; Weintraub *et al.*, 2010; Piray *et al.*, 2014; Erga *et al.*, 2017). These can have devastating personal, financial and social consequences, contributing to the breakup of families and loss of life savings (Leroi *et al.*, 2012*b*; Bartlett, 2013; Okai *et al.*, 2013; Stenberg, 2016; Erga *et al.*, 2017).

The more severe forms of behaviour, meeting formal diagnostic criteria for a ‘disorder’, are reported at a prevalence of ∼15–25% of the Parkinson’s disease population (Weintraub *et al.*, 2010; Baig *et al.*, 2019). However, less severe forms (impulse control behaviours, ICBs), are likely to be more common, with estimates ranging up to 58.3% (Stenberg, 2016). The difference in reported prevalence most likely reflects a recent shift in focus from categorical assessments of these behaviours to a more dimensional approach, differences in definitions, assessment methodology and sociocultural characteristics of the study sample (Callesen *et al.*, 2013; Sharma *et al.*, 2015; Vela *et al.*, 2016; Antonini *et al.*, 2017; Molde *et al.*, 2018; Baig *et al.*, 2019; Evans *et al.*, 2019).

Parkinson’s disease itself is not considered to carry increased risk for development of ICBs (Antonini *et al.*, 2011). Indeed, healthy controls have been shown to have levels of ICB equivalent to newly diagnosed Parkinson’s disease patients (Antonini *et al.*, 2011; Weintraub *et al.*, 2013). Rather, ICDs in Parkinson’s disease are considered to develop in response to dopamine agonist treatment and interact with underlying Parkinson’s disease pathophysiology and possibly personality traits to manifest as particular behavioural phenotypes (Weintraub *et al.*, 2013; Garcia-Ruiz *et al.*, 2014; Sharma *et al.*, 2015; Houeto *et al.*, 2016; Dawson *et al.*, 2018; Baig *et al.*, 2019). In particular, ICDs have been associated with the use of non-ergolinic oral dopamine agonists, such as rasagiline, ropinirole and pramipexole (Garcia-Ruiz *et al.*, 2014; Weintraub *et al.*, 2015), but also with the use of ergoline-derivatives such as cabergoline (Weintraub *et al.*, 2010), although no straightforward dose-dependent relationship, across individuals, has yet been demonstrated (Ambermoon *et al.*, 2011; Corvol *et al.*, 2018; Dawson *et al.*, 2018).

Several questions about vulnerability to the development of Parkinson’s disease ICDs remain. For instance, it remains unclear why only a proportion of patients with Parkinson’s disease develop ICDs (Houeto *et al.*, 2016). Nor has it proven possible to predict with confidence who might be particularly vulnerable. Several possible mechanisms that might contribute to ICDs have been considered including: dysfunction of reward signalling pathways; impaired learning from negative feedback (losses or punishments); enhanced novelty seeking; reduced willingness to wait for rewarding outcomes such that patients have a preference for smaller, immediate rewards over larger, delayed rewards (enhanced delay discounting); rapid decision-making without sufficient evidence (reflection impulsivity); reduced inhibition; and increased risk taking under conditions of ambiguity (for reviews see Basar *et al.*, 2010; Dalley *et al.*, 2011; Napier *et al.*, 2015; Voon *et al.*, 2017; Weintraub and Mamikonyan, 2019). However, there is no general consensus on which of these is most important, and it remains a real possibility that several different mechanisms might contribute since dopamine has many different effects on brain function.

Here we focus on one potential factor, hypothesizing that Parkinson’s disease patients with a history of ICDs are hypersensitive to potentially rewarding outcomes. Dopamine has long been established as having a crucial role in both reward and motivational pathways (Wise, 2004; Salamone and Correa, 2012). Recent investigations in Parkinson’s disease have revealed that it might be possible to measure reward sensitivity using oculomotor indices: either pupillary dilatation in anticipation of rewards or saccadic velocity to visual targets associated with rewards (Bijleveld *et al.*, 2009; Knapen *et al.*, 2016; Koelewijn *et al.*, 2018). Such studies have shown that reward sensitivity is enhanced in patients with Parkinson’s disease when ON dopaminergic medication compared to when OFF these drugs (Manohar and Husain, 2015; Manohar *et al.*, 2015). Furthermore, some Parkinson’s disease patients with apathy—another common neuropsychiatric syndrome (Pagonabarraga *et al.*, 2015)—have blunted sensitivity to rewards as indexed by pupillary dilatation (Muhammed *et al.*, 2016).

The results of several studies now also point to the possibility that dysfunctional reward processing might contribute to the emergence of ICDs in humans. Impulsive healthy individuals are hypersensitive to monetary rewards and tend to prefer small immediate rewards over larger but temporally delayed rewards (Housden *et al.*, 2010; Voon *et al.*, 2011; Leroi *et al.*, 2013; Claassen *et al.*, 2015). Similarly, Parkinson’s disease patients with ICD (PD+ICD) exhibit stronger temporal discounting of monetary rewards, compared to Parkinson’s disease cases without ICD (PD-no-ICD) (Housden *et al.*, 2010; Voon *et al.*, 2010). Furthermore, PD+ICD patients also have significant cortical thinning and altered resting-state connectivity in brain areas involved in reward processing, including ventromedial prefrontal cortex, ventral striatum and amygdala (Biundo *et al.*, 2015; Tessitore *et al*., 2016; Imperiale *et al*., 2018), potentially caused by drug-induced overstimulation of the reward network (Cilia *et al.*, 2008). In contrast, insensitivity to reward has been shown to be an important component of apathy in Parkinson’s disease and basal ganglia disorders (Adam *et al.*, 2013; Martinez-Horta *et al.*, 2014; Le Bouc *et al.*, 2016; Le Heron *et al.*, 2018). Neuroimaging studies of patients with apathy also implicate dysfunction in frontostriatal circuits (Le Heron *et al.*, 2018).

In the current study we used a relatively simple oculomotor paradigm that imposes low demands on decision-making and working memory but potentially provides a relatively pure signal of reward sensitivity. This task was deployed to investigate whether heightened reward sensitivity might occur in Parkinson’s disease patients with a history of ICD. Furthermore, by testing individuals ON and OFF their dopaminergic medication we were able to quantify the effect of dynamic dopamine changes on reward sensitivity in Parkinson’s disease patients with and without ICD. Finally, we also assessed the relationship between impulsivity and apathy in Parkinson’s disease. We hypothesized that pupil dilatory response to forthcoming monetary reward cues and saccadic vigour to targets associated with reward would be greater for larger incentives in both PD+ICD and PD-no-ICD groups, that this reward sensitivity would be heightened while ON dopamine and further enhanced in PD+ICD compared to PD-no-ICD patients. While the first two predictions were verified for pupillary response, it transpired that the latter was not. Instead, we found that PD+ICD cases showed heightened sensitivity to reward both ON and OFF medication, unlike in PD-no-ICD cases who demonstrated increased reward sensitivity ON dopaminergic medication compared to OFF.

## Materials and methods

### Participants

In accordance with the Declaration of Helsinki, informed written consent was obtained from all participants and this study was approved by the local ethics committee. Participants were informed that they would receive bonus monetary rewards according to their performance on the experimental task in addition to compensation for the time spent and reimbursement for travel costs. Patients with Parkinson’s disease were recruited from clinics in the Thames Valley area. Parkinson’s disease patients with impulse control disorder were identified using the Questionnaire for Impulsive-Compulsive Disorders in Parkinson’s Disease (QUIP-Anytime-PD-Short and QUIP-Current-PD-Short; Weintraub *et al.*, 2009). ICD-positive patients then underwent a semi-structured interview with the consultant neurologist to clinically phenotype these patients and further identify their impulsive behaviours. In addition, the Parkinson’s Impulse-Control Scale (PICS), a clinician-rated scale based on semi-structured interview was used to measure the frequency and impact of a range of ICDs (Okai *et al.*, 2016). These interviews were conducted by two clinicians who had been trained by a neuropsychiatrist with a special interest in movement disorders.

Data from 49 patients with a clinical diagnosis of idiopathic Parkinson’s disease are presented here. Twenty-five patients with ICD behaviours (PD+ICD) were recruited to take part in this study (14 males; 23 right-handed). Two patients were not included in the analysis because eye tracking data could not be obtained due to technical difficulties; therefore, the data from 23 PD+ICD cases are presented here. Twenty-six Parkinson’s disease cases (19 male; 21 right-handed) without impulse-control behaviours (PD-no-ICD) were included in the study. At the time of testing, none of these patients reported a history of or current diagnosis of ICD. Thirty-one elderly healthy controls were also tested. The performance on the task of PD-no-ICD and healthy controls was previously reported in Muhammed *et al.* (2016).

Despite often co-occurring in the same patient, it has been proposed that ICBs such as dopamine dysregulation syndrome, and to a lesser extent hobbyism and punding, may have different underlying physiological substrates from ICDs (Evans *et al.*, 2004; Gallagher *et al.*, 2007). None of the PD+ICD cases here had isolated dopamine dysregulation syndrome, hobbyism or punding; all had other manifestations of ICD. For this reason, patients with current and previous ICDs or sub-syndromal ICBs were grouped into a single category in this study.

All 23 PD+ICD patients tested were established on dopaminergic medication (levodopa and/or dopamine agonists). Eleven PD+ICD patients were on levodopa only, none were on only dopamine agonists and 12 were on a combination of both (Supplementary Table 2). Patients were tested ON and OFF their dopaminergic medication in two counterbalanced sessions. For the ON medication session, patients took their dopaminergic medication as per their normal routine. OFF medication, we used a practically defined ‘OFF’ state whereby patients refrained from taking their morning dose before the morning session. This overnight withdrawal was consistent across patients in both groups. Means for time since last dose in both groups are shown in Table 1.

**Table 1:** Demographics of PD+ICD and PD-no-ICD groups

	PD+ICD	PD-no-ICD	PD+ICD versus PD-no-ICD
	Mean (SD)	Mean (SD)	*P*-value
*n*	23	26	
Age, years	63.70 (7.56)	67.19 (5.92)	0.076
Gender	12 male	19 male	0.151
Average age at diagnosis	55.09 (7.25)	62.33 (7.41)	<0.01*
Disease duration, years	8.71 (4.25)	4.87 (4.09)	<0.05*
Symptom duration, years	10.97 (4.71)	7.34 (4.45) (*n* = 22)	<0.05*
Apathy, LARS Total	−20.09 (6.16)	−22.23 (8.65)	0.299
Depression, BDI	12.26 (5.84)	13.00 (7.10)	0.695
Cognitive screen, MoCA	27.39 (2.55)	27.77 (1.95)	0.560
Anhedonia, SHAPS	48.61 (4.55)	48.35 (5.69)	0.865
Anhedonia, TEPS Total	61.52 (8.99)	60.65 (7.03)	0.707
BIS/BAS: BIS	19.96 (4.76)	12.54 (2.49)	<0.001*
BIS/BAS: BAS Drive	9.43 (1.67)	10.65 (2.35)	<0.05*
BIS/BAS: BAS Reward Responsiveness	14.57 (2.41)	9.35 (2.15)	<0.001*
BIS/BAS: BAS Fun-Seeking	10.30 (1.79)	9.77 (2.41)	0.387
DASS Total	27.35 (16.82)	21.88 (15.10)	0.237
UPDRS Total	59.09 (31.43)	37.04 (15.37)	<0.05*
UPDRS Part 1	14.17 (7.55)	7.46 (4.84)	<0.05*
UPDRS Part 2	15.83 (9.45)	9.54 (4.36)	<0.05*
UPDRS Part 3_ON	24.22 (16.94)	18.62 (9.38)	0.169
UPDRS Part 3_OFF	33.65 (16.61)	27.08 (9.61)	0.105
UPDRS Part 3 ON minus OFF	9.43 (5.24)	8.46 (4.18)	0.473
UPDRS Part 4	4.87 (5.04)	1.42 (2.64)	<0.05*
Hoehn and Yahr stage	1.83 (0.83)	1.31 (0.62)	<0.05*
Hours since last dose: ON versus OFF	2.57 (±1.16) versus 14.22 (±2.01)	2.56 (±2.34) versus 14.28 (±4.3)	0.976 versus 0.953
Levodopa equivalent dose, mg/24 h	716.32 (324.94)	497.15 (335.97)	<0.05*
Dopamine agonist equivalent dose, mg/24 h	126.81 (153.31)	75.84 (137.49)	0.226

Values in parentheses represent standard deviations. BDI = Beck Depression Inventory; BIS/BAS = Behavioural Inhibition Scale/Behavioural Activation Scale; DASS = Depression Anxiety Stress Scales; LARS = Lille Apathy Rating Scale; MoCA = Montreal Cognitive Assessment; SHAPS = Snaith-Hamilton Pleasure Scale; TEPS = Temporal Experience of Pleasure Scale; UPDRS Part 1 = Non-Motor Aspects of Experiences of Daily Living, Part 2 = Motor Aspects of Experiences of Daily Living, Part 3 = Motor Examination (ON Dopamine), Part 4 = Motor Complications.

* Significant result.

In the PD-no-ICD group, 15 patients were on levodopa only, and 11 were on a combination of levodopa and dopamine agonists. We found no evidence that any of the 15 PD-no-ICD patients only currently on levodopa had previously taken dopamine agonists. It is, of course, possible that this could reflect reverse causation in the recruitment of patients, that the decision to start an agonist might have been avoided in subjects with greater impulsivity or patients with a high risk of developing ICDs. However, we have no data on this aspect of clinical decision-making for these individuals.

### Demographic and clinical measures

PD+ICD cases were administered the short form of the QUIP known as the QUIP-S. This is a self-report questionnaire, consisting of two parts, QUIP-anytime and QUIP-current (Weintraub *et al.*, 2009). The cut-off to indicate ICD in Parkinson’s disease is one or more affirmative response to any question, in either the current or anytime QUIP-S, representing the presence of the ICD behaviour for a period of at least 4 weeks. The self-report UPPS-P (Urgency, Premeditation, Perseverance, Sensation Seeking, Positive Urgency) Impulsive Behaviour Scale was used to assess personality traits which underlie impulsive behaviour (Whiteside and Lynam, 2001). The BIS/BAS scale (Behavioural Inhibition System and Behavioural Activation System) was also used to assess behavioural inhibition and behavioural activation, two motivational systems which regulate behaviour and affect. The BIS/BAS scale consists of three subscales relating to behavioural activation: drive, fun seeking and reward responsiveness, and one subscale relating to behavioural inhibition (Carver and White, 1994).

Apathy was assessed with the Lille Apathy Rating Scale (LARS), a semi-structured clinical interview which has previously been validated in Parkinson’s disease (Sockeel *et al.*, 2006). A score threshold of ≥−22 was set to indicate borderline, or mild apathy, and ≥−16 to indicate clinical, or severe apathy. Cognitive impairment was assessed and screened using the Montreal Cognitive Assessment (MoCA) (Nasreddine *et al.*, 2005) and depression using the Beck Depression Inventory (BDI-II; Beck *et al*., 1996). Degree of anhedonia was assessed using the Temporal Experience of Pleasure Scale (TEPS) (Gard *et al.*, 2006), and the Snaith-Hamilton Pleasure Scale (SHAPS) (Snaith *et al.*, 1995). Parkinson’s disease symptom severity was assessed using the Movement Disorder Society Unified Parkinson’s Disease Rating Scale (MDS-UPDRS) and disease stage was assessed using the Hoehn and Yahr scale (Goetz *et al.*, 2008). None of the participants had a history of autonomic dysfunction or major psychiatric illness.

### Experimental paradigm

**Figure 1:**
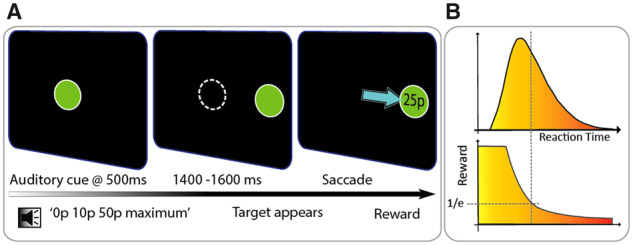
**Experimental paradigm.** (**A**) Participants were informed of the maximum reward available at the start of each trial with an auditory cue initiating 500 ms after trial onset: ‘0p/10p/50p maximum’. Subsequent to a randomized variable fore-period of 1400, 1500 or 1600 ms the central ﬁxation disc disappeared concurrently with the appearance of a new peripheral target disc. Participants’ rewards varied according to reaction time, and the obtained reward was displayed within the peripheral target disc in pence (e.g. 25p). (**B**) The absolute reward value obtained varied with reaction time, but importantly was dynamically adjusted according to each participant’s mean reaction time at any point during the experiment. Rewards obtained for each trial were calculated using an adaptive exponential fall-off based on the mean reaction time of the preceding 20 trials and, dependent on performance, participants received a proportion of the maximum amount on offer. This adaptive procedure allowed difﬁculty level to be kept constant over the experiment and crucially meant that all participants received the same overall reward amount. Therefore, it prevented some patients earning less as they progressed through the task while maintaining equal extrinsic incentivization.

Eye position and pupil diameter were monitored using an infrared eye tracker (Eyelink 1000, SR Research) while participants made eye movements toward targets presented on a computer screen. They were instructed to make quick, direct eye movements and were told that the faster that they looked at peripheral targets, the greater a proportion of the reward on offer they would receive (Fig. 1A). In each trial, participants were first asked to fixate on a disc target at the centre of the screen measuring 4° in diameter. After 500 ms a recorded voice stated the maximum possible reward for that particular trial: ‘0p/10p/50p maximum’. Subsequently, following a variable period of 1400, 1500 or 1600 ms the central fixation target disappeared and synchronously a target disc appeared randomly either to the left or the right of the screen along the horizontal meridian, at 11° eccentricity.

In each trial, participants received a proportion of the maximum reward on offer dependent on their saccadic performance. The absolute amount of reward they received varied with reaction time, but importantly this amount was dynamically adjusted using an ‘adaptive’ exponential fall-off based on average reaction time of the 20 previous trials (Fig. 1B). This allowed a consistent difficulty level to be maintained throughout the experiment while factoring in different participants’ baseline reaction times and fatigue rates. Moreover, this procedure meant that each participant received equal reward amounts across the experiment, therefore differences in performance between the groups could not be attributed to differences in overall rewards obtained. At the end of each trial, the obtained reward for that trial was displayed within the peripheral target disc in pence. This visual feedback ensured that all participants were equally extrinsically motivated by the rewards that they earned during the task.

In all trials a saccade to the peripheral target was required in order to move to the next trial, including in the 33% of trials in which there was no reward stimulus and no reward on offer (the 0p condition).

All participants performed five experimental blocks consisting of 54 trials and were given a 5-min break between the third and fourth blocks. Therefore, they each performed 270 trials in total, 90 for each reward condition. Disc luminance and ambient room lighting was consistent across all trials to avoid differentially affecting pupil dilation. If a reward of 10p or greater was earned, an audible bell sound was played, and for rewards of 30p or greater the sound of a cash register was played concurrently with the appearance of the visual feedback. For earned rewards of less than 10p no sound was played but for 0p a low frequency buzzing sound was played. Behavioural indicators of task performance included saccadic speed (mean peak velocity) and saccadic variability [standard deviation (SD) of saccadic amplitude].

### Eye tracker data recording

Participants sat 60 cm from a 21′′ CRT computer screen (1024 × 768 pixels; 100 Hz refresh rate) in a dimly lit room. Visual stimuli were presented using MATLAB (The MathWorks) and Psychophysics Toolbox on a Microsoft Windows PC. The frame mounted infrared eye tracker monitored left eye position at a sampling rate of 1 kHz and a 9-point calibration was performed. The Eyelink computer measured eye movements online and fed back into the presentation computer to provide immediate feedback.

Analysis of the eye tracking data was performed using standard Eyelink criteria, with a velocity and acceleration threshold for saccade initiation. Saccades were defined as eye movements >2° and were considered to be complete when they landed within a 5° radius from the target centre. Reaction times were calculated as the time when the target first appeared to the time of saccade completion and peak saccadic velocity was calculated as the maximum speed of the eye position trace in windows of 3 ms, measured for the first saccade that landed outside the central fixation disc. Pupil trajectories which were lost due to blinking were interpolated up to a maximum of 500 ms, trials with larger gaps were discarded. Pupil dilation was measured as the proportional change from the average baseline pupil size. For each trial, recordings were time locked to the onset of the reward cue and normalized by subtracting the baseline pupil size at 0 ms. A moving average smoothing window of 10 ms, then 100 ms was applied.

### Eye tracker data analysis

Analysis of pupillary and saccadic data was performed using split-level ANOVA, with within-subjects factors of Drug (ON, OFF) and Reward (0p, 10p, 50p), and between-subjects factor of Clinical Group (PD+ICD, PD-no-ICD). For each saccadic and pupillary variable a new variable reflecting reward sensitivity was calculated as the difference in mean proportional pupil change between the 50p and 0p reward conditions (50p − 0p) and further analyses were performed using repeated measures ANOVA, with a within-subjects factor of Drug (ON, OFF) and a between-subjects factor of Clinical Group (PD+ICD, PD-no-ICD). Significance in the pupil trace figures was calculated using a permutation analysis whereby *P*-values were calculated for every time point after 600 ms. Where appropriate, statistics were reported with Greenhouse-Geisser corrections, to account for non-sphericity in the data. If significant interactions were observed, corrected pairwise comparisons were made using Bonferroni *post hoc* tests. Significance was taken as *P*-values of < 0.05. For non-parametric questionnaire data, Spearman Rank correlations were performed, and Pearson correlations were utilized for parametric testing of behavioural eye tracking data. Bonferroni corrections for multiple correlations were performed. Statistics were performed using MATLAB, SPSS and JASP.

### Data availability

Applications for deidentified demographic data can be made to the Oxford Parkinson’s Disease Centre. See https://www.opdc.ox.ac.uk/external-collaborations for further information.

## Results

### General characteristics of patient groups

There were no significant differences between the PD+ICD and PD-no-ICD groups in age, cognitive impairment (MoCA), depression (BDI) or anhedonia (SHAPS/TEPS), or anxiety (Depression Anxiety Stress Scale, DASS) (Table 1). However, PD+ICD patients had significantly longer disease and symptom duration than PD-no-ICD, were younger at diagnosis and had higher levodopa equivalent dose (LEDD). There was no significant difference between PD+ICD and PD-no-ICD groups in dopamine agonist levodopa equivalent dose (DA-LEDD) and there were no significant correlations between impulsivity (UPPS-P) or apathy (LARS) with duration of symptoms, duration of disease, or age.

Furthermore, the two groups differed in UPDRS Total [unpaired *t*(31.073) = 3.057, *P = *0.005] and in the subscales; UPDRS Part 1 [unpaired *t*(36.647) = 3.649, *P = *0.001], Part 2 [unpaired *t*(30.110) = 2.927, *P = *0.006], Part 4 [unpaired *t*(32.311) = 2.942, *P = *0.006], and Hoehn and Yahr stage [Mann-Whitney U-test (4.887) = 195.00, *P = *0.021], reflecting overall greater Parkinson’s disease symptom severity in PD+ICD.

All patients in the PD+ICD group had experienced at least one ICD anytime in their life, for at least a 4-week period, which was highlighted during a clinical assessment leading to a formal diagnosis of ICD (Fig. 1A and Supplementary Table 1). Current (at time of data collection) prevalence of ICDs was less than the anytime rates because in most cases the patient’s dopaminergic medication dose had been reduced to improve ICD symptoms. Eleven of the PD+ICD patients were currently taking only levodopa and 12 were taking a combination of levodopa and dopamine agonists. Of the 11 only taking levodopa, nine had previously taken dopamine agonists before undergoing dopamine withdrawal to account for the emergence of impulsive behaviours (Supplementary Table 2). When comparing ICDs, 69.6% (16/23) of PD+ICD patients reported multiple ‘anytime’ ICDs, compared to 44.5% (10/23) who currently had multiple ICDs.

### Comorbidity of impulse control disorders and apathy

There was significant comorbidity of apathy and impulsivity. Of PD+ICD patients, 78.3% were either borderline (43.5%) or clinically (34.8%) apathetic. This is a higher proportion than in the PD-no-ICD group in which 46.2% were either borderline (11.5%) or clinically (34.6%) apathetic as measured by the LARS clinical interview. There were no significant differences between PD+ICD and PD-no-ICD in LARS total, or in the motivational domain of intellectual curiosity (cognitive apathy). Furthermore, the groups did not differ significantly in the self-awareness component of the LARS. However, they did differ significantly in two of the three motivational domains: action initiation (behavioural apathy) with the PD-no-ICD group scoring significantly better (higher levels of action initiation) than PD+ICD [Mann-Whitney U-test (49.28) = 184.0, *P = *0.020], and emotional apathy with PD+ICD exhibiting greater levels of emotional apathy than PD-no-ICD [Mann-Whitney U-test (49.35) = 198.5, *P = *0.042].

### No difference in baseline dopaminergic effect in impulse control disorders

We first examined baseline pupil size to ensure it could not account for the reported group differences on reward sensitivity. Baseline pupil size for each patient was measured as the mean pupil diameter (in arbitrary units) across all experimental reward conditions (0p, 10p, 50p) at the beginning (0 ms) of each trial, at the time of onset of the auditory reward cue. PD+ICD patients had greater mean pupil sizes than PD-no-ICD [drug × group (2 × 2) repeated measures ANOVA, main effect of group, *F*(1,47) = 7.058, *P = *0.011]. Across both groups, as reported previously in Parkinson’s disease (Muhammed *et al.*, 2016), baseline pupil size was larger ON than OFF dopaminergic medication [main effect of drug, *F*(1,47) = 29.694, *P < *0.001]. There was no significant difference in this dopaminergic effect between the groups, i.e. baseline pupil size ON dopamine minus OFF dopamine (interaction between drug and group, *P = *0.909). Note that our interest in this study is in the subsequent impact of the reward incentives on changes from baseline–pupil reward sensitivity, discussed below. The key points from the baseline pupil size analysis show that PD+ICD patients had larger pupils, so if anything, they have less potential dynamic range for further dilation to reward, and all patients have bigger pupils ON dopamine, again meaning that there is less potential dynamic range for more dilation.

### Differential dopaminergic effects on pupil reward sensitivity

Pupillary modulation in response to monetary reward was calculated as the mean proportional difference in pupil size from the baseline pupil diameter at the beginning of each trial. As previously (Muhammed *et al.*, 2016), the relevant epoch for reward related pupil response was taken between 1400 ms and 2400 ms after the auditory reward cue. We were interested in whether pupil dilatation was related to the potential reward on offer in each trial, and whether there were differences in pupillary dilation between PD+ICD and PD-no-ICD. Each patient’s ‘pupillary reward sensitivity’ was calculated as the difference in mean proportional pupil change between the 50p and 0p reward conditions across the time epoch described above. Greater difference between 50p and 0p reflects greater reward sensitivity.

**Figure 2:**
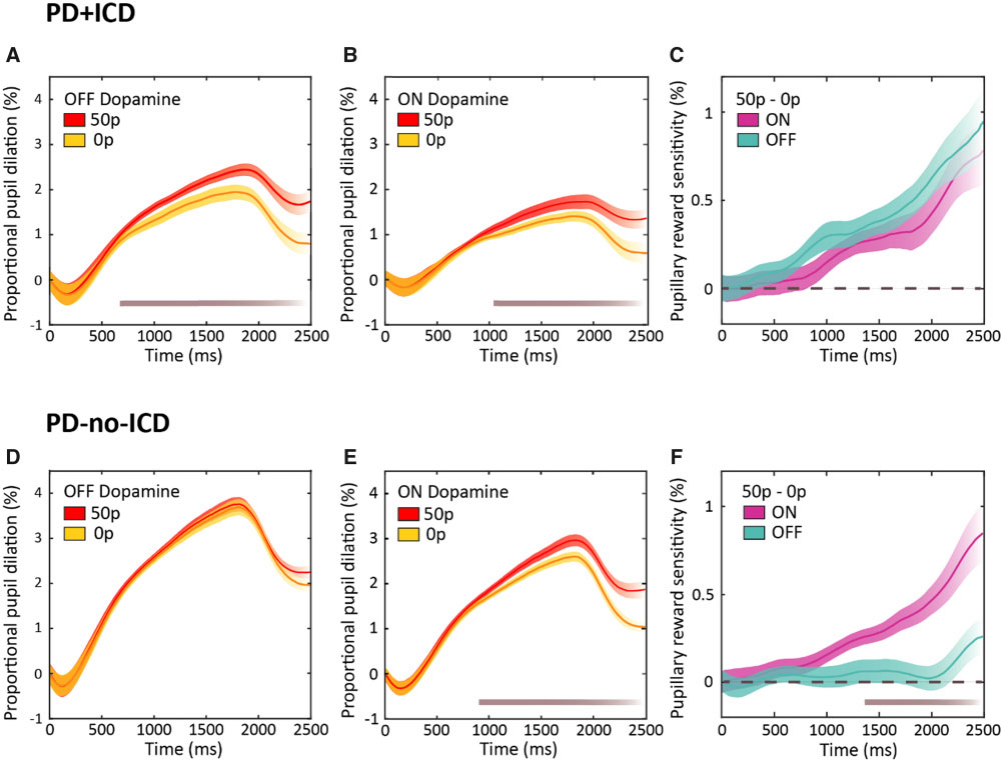
**Dynamics of pupil response on low and high reward trials.** (**A**) Mean pupillary trace in PD+ICD OFF dopaminergic medication following onset of auditory reward cue at 0 ms until trial end at 2500 ms. Pupil dilation was calculated as the proportional change from mean baseline before onset of the stimuli. A significant difference between pupil dilation in response to the 50p reward (red) and 0p reward cue (yellow) present from ∼630 ms (*P *<* *0.05), depicted by the grey bar at the *bottom* of the plot. (**B**) Mean pupillary trace in PD+ICD ON dopaminergic medication. A significant difference between the 50p reward and 0p reward cue present from ∼1020 ms (*P *<* *0.05) only. (**C**) Mean pupil dilation reward sensitivity, calculated as proportional pupil change in response to the 50p reward cue minus change to the 0p condition, in PD+ICD ON (turquoise) and OFF (purple) dopamine. No significant difference was found between reward sensitivity ON and OFF at any time point. (**D**) Mean pupillary trace in PD-no-ICD OFF dopaminergic medication. No significant difference was found between 50p and 0p at any time point. (**E**) Mean pupillary trace in PD-no-ICD ON dopaminergic medication. A significant difference between the 50p reward and 0p reward cue present from ∼890 ms (*P *<* *0.05). (**F**) Mean pupil dilation reward sensitivity, in PD-no-ICD ON and OFF dopamine. A significant difference between reward sensitivity ON and OFF dopamine present from ∼1420 ms (*P *<* *0.05). Shaded area represents standard error across subjects after subtracting the mean.

**Figure 3:**
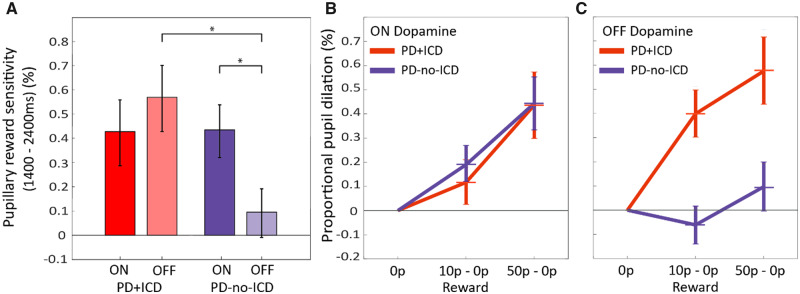
**Pupillary responses to rewards in PD+ICD and PD-no-ICD.** (**A**) Mean proportional pupil reward sensitivity (mean pupillary change for 50p condition minus 0p condition) for PD+ICD and PD-no-ICD ON and OFF dopaminergic medication. In PD-no-ICD withdrawing dopamine significantly reduced pupillary reward sensitivity but this was not observed in PD+ICD. The distribution of individual subject’s data is provided in the Supplementary material. (**B**) Proportional pupillary dilation as a function of reward level in PD+ICD (red) and PD-no-ICD (blue) ON dopaminergic medication, taken as the mean pupil dilation between 1400 ms and 2400 ms. Changes have been normalized to the 0p baseline to demonstrate the relationship between reward sensitivity slopes. (**C**) Proportional pupillary dilation as a function of reward level in PD+ICD (red) and PD-no-ICD (blue) OFF dopaminergic medication. In PD+ICD being ON dopamine reduced reward sensitivity whereas in PD-no-ICD dopamine increased reward sensitivity. *P <* *0.05.

In both PD+ICD and PD-no-ICD, pupils dilated more in response to increasing monetary reward [repeated measures reward × drug × group (3 × 2 × 2) three-way ANOVA, main effect of reward condition on mean pupil change over the time epoch of interest, *F*(2,94) = 23.927, *P < *0.001] (Figs 2 and 3A). In addition, there was a significant three-way interaction between drug condition, reward and group [*F*(2,94) = 5.692, *P = *0.005] reflecting differences in the dopaminergic effect on pupil reward sensitivity in the two groups. Furthermore, there was a main between-subjects effect of group (PD+ICD versus PD-no-ICD) revealing overall smaller pupil responses to reward in PD+ICD [*F*(1,47) = 11.129, *P = *0.002], as well as a main effect of drug condition with greater overall pupil response when OFF dopamine in both groups [*F*(1,47) = 14.772, *P < *0.001]. There were no two-way interactions.

These results indicate that overall there was less pupillary dilation while ON dopamine. However, this does not reflect reward insensitivity. Rather, dopamine has a mydriatic effect on the pupil, increasing baseline pupil size and hence allowing less scope for further dilatory response to reward. Our primary interest here is relative reward sensitivity. Despite reduced overall dynamic pupil change while ON dopamine, reward sensitivity (i.e. greater pupil dilation for greater reward on offer) effects were observed in both groups.

To decompose the three-way interaction, we next examined the drug and reward effects separately within each group, performing a reward × drug (3 × 2) repeated measures ANOVA. In the PD-no-ICD group, being ON dopamine led to a significant increase in reward-related pupil dilation [interaction between drug and reward, *F*(2,50) = 5.336, *P = *0.008; Fig. 3A]. Furthermore, reward increased pupil dilation [*F*(2,50) = 7.044, *P = *0.002], whereas dopamine reduced it [main effect of drug, *F*(1,25) = 7.594, *P = *0.011]. The interaction in the PD-no-ICD group is indicative of a steeper reward sensitivity slope ON medication compared to OFF (Fig. 3B and C).

By contrast, In PD+ICD, there was no significant difference in reward sensitivity ON versus OFF dopamine [reward × drug interaction, *P =* 0.145; Fig. 3A]. Overall, just as for the PD-no-ICD group, being ON dopamine reduced pupil dilation [main effect of drug, *F*(1,22) = 7.585, *P = *0.012] whereas reward increased it [main effect of reward, *F*(2,44) = 17.827, *P < *0.001]. Bonferroni *post hoc* pairwise comparisons between the three reward levels (0p versus 10p, 10p versus 50p and 0p versus 50p) demonstrated significant differences in pupillary reward response between each level (*P *<* *0.01). In the PD+ICD group, there was also no significant difference between the sensitivity slopes ON and OFF dopamine (Fig. 3B and C). Overall reward sensitivity was far less in PD-no-ICD, as indicated by the shallower slopes of pupil response between the 0p and 50p conditions, compared to the PD+ICD group (Fig. 3B and C).

Next, we compared elderly healthy controls to each group of Parkinson’s disease patients and found that the only significant difference lay in the comparison with PD-no-ICD cases OFF dopaminergic medication: in this state, but not in the ON state, these patients showed significantly reduced reward sensitivity compared to healthy controls [*F*(1,55) = 8.643, *P = *0.005]. For PD+ICD cases, there was no significant difference compared to healthy controls, either in the ON or OFF state. Analysis of the difference in pupillary reward sensitivity ON versus OFF dopaminergic medication (ON − OFF) demonstrated a significant difference between PD+ICD and PD-no-ICD groups [unpaired *t*(47) = −2.338, *P = *0.024]. These findings highlight that, quite different to our initial hypothesis, PD+ICD cases do not in fact show enhanced reward sensitivity ON dopaminergic medication, but rather show enhanced sensitivity to reward both ON and OFF, with no significant difference between these states, unlike PD-no-ICD patients who show greater sensitivity ON as previously reported for patients with Parkinson’s disease (Muhammed *et al.*, 2016).

### Potential confounding factors

As noted earlier, overall there were no significant differences between the PD+ICD and PD-no-ICD groups in age, cognitive impairment (MoCA), depression (BDI), anhedonia (SHAPS/TEPS), or anxiety (DASS) (Table 1). Furthermore, there was no significant difference between PD+ICD and PD-no-ICD groups in dopamine agonist levodopa equivalent dose (DA-LEDD). However, PD+ICD cases had significantly longer disease and symptom duration, were younger at diagnosis and had higher overall LEDDs than PD-no-ICD patients.

To investigate the role of potential confounds more closely, we assessed the effects of: age, disease severity, disease duration, currently active versus previously active ICD, current dopamine agonist therapy (with levodopa) versus levodopa therapy alone, and cholinergic medications

There were no significant correlations between age and pupil reward sensitivity ON or OFF dopamine, or between age and the effect of dopamine on reward sensitivity (RS ON −OFF). PD+ICD cases had higher symptom severity scores, as measured by the UPDRS (Table 1). We therefore tested whether the difference in pupil response to reward was influenced by differences in Parkinson’s disease symptom severity. Across all patients, the effect of dopamine on pupil reward sensitivity (RS ON − OFF) did negatively correlate significantly with UPDRS Part 2 (*S^r^* = −0.359, *P = *0.011), the motor symptoms section; there was a trend towards correlation with UPDRS Part 1 which assesses non-motor symptoms (*S^r^* = −0.260, *P = *0.071). Total UPDRS also trended towards significance (*S^r^* = −0.264, *P = *0.066). Within each group, there were no significant correlations; this might reflect sample size. Overall, patients with more pronounced motor symptoms (UPDRS Part 2), therefore had weaker effects of dopamine on reward sensitivity, i.e. a smaller increase in reward sensitivity when ON compared to when OFF dopaminergic medication.

We next asked whether this correlation in drug effect was driven by being ON or by being OFF drug. However, no significant correlations were found between UPDRS total or subscales and pupillary reward sensitivity either ON or OFF dopamine in the PD+ICD group or the whole Parkinson’s disease population ON or OFF dopamine. Thus, it was specifically the difference in response to reward ON versus OFF dopamine that was negatively correlated with motor symptom severity. There was no significant effect of disease duration (from time of diagnosis). However, patients with longer prodrome periods (difference in time between symptom onset and disease diagnosis) were more likely to have larger effects of dopamine on pupillary reward sensitivity (Supplementary material).

Next, we performed a *post hoc* subgroup analysis to assess whether there were differences in pupil reward sensitivity in PD+ICD patients with currently active (*n *=* *15) versus previous ICD (*n *=* *8) symptoms and found no significant differences between these groups (Supplementary material). Furthermore, we grouped PD+ICD patients with current or past ICDs who take dopamine agonists in addition to levodopa (*n *=* *12) with those who only take levodopa (*n *=* *11). We found no significant differences between these subgroups in terms of proportional pupil change reward sensitivity ON dopamine. However, PD+ICD patients currently on agonists had significantly greater pupil response to reward OFF dopamine [*F*(1,21) = 5.368, *P = *0.031], mean of ON and OFF dopamine [*F*(1,21) = 5.814, *P = *0.025] but crucially not in the effect of dopamine on reward sensitivity (RS ON-OFF) [*P = *0.380] (Supplementary material).

To assess whether the overall PD+ICD result of greater reward sensitivity in the OFF condition compared to PD-no-ICD cases could all be attributed to the PD+ICD cases on dopamine agonists, we performed another *post hoc* analysis in which we excluded all PD+ICD patients who were currently taking dopamine agonists. These patients (*n *=* *11) were on levodopa and were compared to the PD-no-ICD cases (*n *=* *26). There were significant main effects of reward [*F*(2,70) = 10.914, *P < *0.001], drug (ON versus OFF) [*F*(1,35) = 11.994, *P = *0.001] and group [*F*(1,35) = 5.607, *P = *0.024] as well as a significant 3-way interaction between reward × drug × group [*F*(2,88) = 3.845, *P = *0.026]. Thus, even when excluding all PD+ICD patients who were currently taking dopamine agonists at the time of testing the main findings reported in the text are preserved, despite a significantly smaller sample size in PD+ICD cases who were not dopamine agonists, but taking only levodopa.

We also examined the effect of LEDD and DA-LEDD (dopamine agonist LEDD). There were no significant correlations between LEDD and pupil reward sensitivity ON, OFF, overall mean or ON-OFF. However, there were significant correlations between DA-LEDD and pupil reward sensitivity OFF (*S^r^* = 0.322, *P = *0.024) and the mean for ON and OFF (*S^r^* = 0.366, *P = *0.010). Thus, increased DA-LEDD predicts increased proportional pupil change OFF medication, and mean overall proportional pupil change, but not ON medication or crucially the difference between ON and OFF.

Although nine of the PD+ICD patients had previously withdrawn from dopamine agonists, and one had tapered their agonist dose by half, it is unlikely that dopamine agonist withdrawal syndrome (DAWS) was a significant factor affecting their reward sensitivity. We obtained the dopamine agonist withdrawal dates for five cases. For these patients the average time of withdrawal before testing on our oculomotor task was 5.4 years (range: 2.5–10 years). While it is possible that ongoing subclinical effects were present, it is quite unlikely that this was a significant factor as DAWS usually does not affect patients for this long. Approximately half of DAWS cases resolve themselves within days or weeks but in more serious cases it can last months or years (Nirenberg, 2013).

Finally, we examined the impact of cholinergic medication use. In the PD+ICD group only two patients were taking cholinergic medication (rivastigmine *n *=* *1, donepezil *n *=* *1). In the PD-no-ICD group, only one patient was currently taking cholinergic medication (trihexyphenidyl). These medications are known to have direct mydriatic effects on the pupil. We reanalysed the data after excluding all patients who were taking cholinergic medication and the overall findings were not affected (Supplementary material).

These analyses suggest that the several potential confounding factors considered here are unlikely to explain the pattern of findings we describe for pupil reward sensitivity in PD+ICD cases.

### Proportional pupil change in response to reward predicts future emergence of impulse control disorders

Because of the pupillary reward sensitivity findings in the PD+ICD group (Figs 2 and 3) we decided to track down patients who had been in our PD-no-ICD group to assess whether any of them had developed ICDs or ICBs in the four to five intervening years since they had initially been studied on this eye tracking paradigm. At the original time of testing, none of the 26 PD-no-ICD patients had self-reported or clinically identified ICD symptoms. For 22 of these patients, ∼4–5 years later (mean = 51 months, SD = 2.86 months) QUIP-anytime and QUIP-current scores were collected. Additionally, two patients from the PD+ICD group had previously been tested on the eye-tracking paradigm before they had developed ICDs, therefore, a similar 4-year follow-up QUIP was available for these patients.

**Figure 4:**
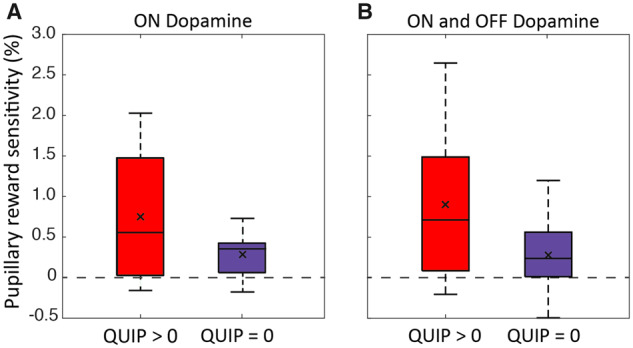
**Pupil reward sensitivity in those who developed a QUIP score > 0.** PD-no-ICD group split based on QUIP-anytime scores collected 4–5 years after behavioural testing with the eye tracking paradigm. (**A**) Mean pupillary reward sensitivity ON dopamine significantly greater in PD-no-ICD with QUIP > 0 compared to PD-no-ICD with QUIP = 0. (**B**) Mean overall pupillary reward sensitivity (ON and OFF dopamine combined) significantly greater in PD-no-ICD with QUIP > 0 compared to PD-no-ICD with QUIP = 0. The line within the box indicates the median; the ‘x’ within depicts the mean.

Of the 24 patients, 14 (66.7%) had a QUIP-anytime score > 0 (mean = 2.79, SD = 2.04), indicating the emergence of ICD behaviours at some point in the last 4–5 years since behavioural testing, lasting for at least 4 weeks (Supplementary Fig. 1B). The 24 patients were subdivided into those who developed ICD (QUIP-anytime > 0; *n *=* *14), and those who did not (QUIP-anytime = 0; *n *=* *10). Pupillary reward sensitivity ON dopamine was greater in the QUIP > 0 group than the QUIP = 0 group [unpaired *t*(22) = −1.834, *P = *0.007; Fig. 4A], with no significant difference OFF dopamine. Overall mean pupillary reward sensitivity (mean of ON and OFF sessions) was also greater in the QUIP > 0 subgroup than the QUIP = 0 subgroup [unpaired *t*(20.8692) = −2.301, *P = *0.032; Fig. 4B].

These results suggest, albeit weakly, that pupil reward sensitivity may be predictive of future development of ICBs in Parkinson’s disease. Importantly, there were no differences between these two groups in apathy (LARS total scores), depression (BDI), anhedonia (TEPS), Parkinson’s disease symptom severity (UPDRS Total), behavioural activation (BAS drive, fun seeking, reward, behavioural inhibition), age, years of symptoms, LEDD or DA-LEDD.

### Saccadic velocity, accuracy and reaction time

The vigour of saccadic movements, quantified by peak velocity, and the motor variability, quantified by standard deviation of saccadic amplitude, are also recognized as markers of motivation (Chen *et al.*, 2013, 2014; Manohar and Husain, 2015; Manohar *et al.*, 2015; Muhammed *et al.*, 2016). We next examined these parameters in the two Parkinson’s disease groups. Mean peak velocity increased with reward [reward × drug × group (3 × 2 × 2) repeated measures ANOVA; main effect of reward, *F*(2,94) = 29.212, *P < *0.001], demonstrating that exogenous monetary reward invigorated saccadic responses across both groups. Peak velocities were actually faster OFF drug [main effect of drug, *F*(1,47) = 12.651, *P = *0.001], as in previous studies (Muhammed *et al.*, 2016). However, saccadic invigoration by reward was modulated by dopamine [interactions reward × drug, *F*(2,94) = 4.669, *P = *0.012] with greater reward sensitivity ON dopamine. There was a trend towards greater invigoration by reward in PD+ICD compared to PD-no-ICD, but this was not significant [reward × group, *F*(2,94) = 2.780, *P = *0.067] and there was no significant three-way interaction [drug × reward × group (*P = *0.675)]. There were no significant differences between the two groups in mean peak velocity or interaction between drug condition and group (Supplementary Fig. 2).

Mean peak velocity does not account for variations in saccadic amplitude, which can influence saccadic velocity with a predictable pattern. The ‘saccadic main sequence’ describes how peak velocity scales with saccadic amplitude; increasing amplitudes are associated with greater peak velocities (Bahill *et al.*, 1975). Reward increased the amplitudes of saccades, making them less hypometric and thereby landing closer to the target [reward × drug × group (3 × 2 × 2) repeated measures ANOVA on saccadic amplitude: main effect of reward, *F*(2,94) = 18.599, *P < *0.001]. This occurred more when ON drug [interaction between reward and drug, *F*(2,94] = 3.632, *P = *0.030]. There was no significant main effect of drug itself on saccadic amplitude and no effects of group or other interactions, indicating that both groups showed similar amplitude effects. Therefore, the increased velocities observed with increasing reward could, in principle, be explained by effects on increased saccadic amplitudes rather than saccadic invigoration by monetary reward.

**Figure 5:**
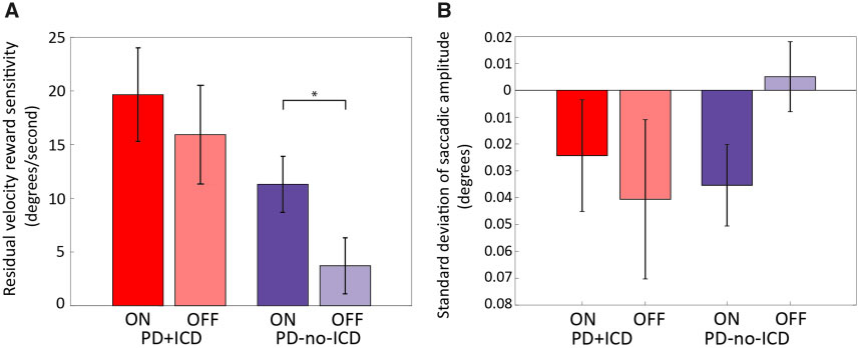
**Saccadic velocity and amplitude ON and OFF dopaminergic medication.** (**A**) Mean peak residual velocity reward sensitivity (mean peak residual velocity for 50p condition − 0p condition) for PD+ICD ON (red) and OFF (light red), and for PD-no-ICD ON (blue) and OFF (light blue). No significant effect of dopamine on residual velocity reward sensitivity was observed in PD+ICD, whereas in PD-no-ICD residual velocity reward sensitivity was significantly greater ON dopamine, compared to OFF. However, PD+ICD did not exhibit greater overall residual velocity or greater reward sensitivity than PD-no-ICD. Furthermore, there was no significant difference between PD+ICD and PD-no-ICD ON or OFF medication although there is a trend towards PD+ICD having greater reward sensitivity OFF dopamine (*P = *0.066). The distribution of individual subject’s data is provided in the Supplementary material. (**B**) Mean standard deviation of saccadic amplitude reward sensitivity in PD+ICD ON (red) and OFF (light red) and PD-no-ICD ON (blue) and OFF (light blue) dopaminergic medication. No difference was found between the reward sensitivity ON and OFF dopamine in PD+ICD whereas in PD-no-ICD there was significantly reduced variability of saccadic amplitude reward sensitivity (error) ON dopamine compared to OFF. Significance indicators reflect pairwise *t*-tests, and are described in the Supplementary material. *P <* *0.05. The distribution of individual subject’s data is provided in the Supplementary material.

To account for this potential effect of amplitude on saccadic velocity, the amplitude of each saccade was factored out by applying a linear regression on the raw saccade data, to obtain the mean peak residual velocities [as previously performed in Muhammed *et al.* (2016); Fig. 5A]. Across the groups, reward sensitivity was preserved after accounting for the amplitude variability [reward × drug × group (3 × 2 × 2) three-way repeated measures ANOVA, main effect of reward, *F*(2,94) = 23.856, *P < *0.001]. Thus, the observed effect of reward cues on peak velocity is not attributable to the increased amplitudes. This analysis revealed no significant main effect of drug, of group or the interaction between reward and group, demonstrating that PD+ICD do not have greater reward sensitivity of velocity residuals or overall faster saccades than PD-no-ICD. There were no other significant two- or three-way interactions, but we note that the overall pattern of results (Fig. 5A) mirrors that for the pupillary reward sensitivity response, with the PD+ICD group demonstrating high reward sensitivity in both the OFF and ON condition, unlike PD-no-ICD cases.

Variability (SD) of saccadic amplitude can be understood as a measure of saccadic precision. Lower amplitude variability reflects greater accuracy of saccadic amplitude while, conversely, greater amplitude variability is indicative of greater saccadic imprecision. According to the speed-accuracy trade-off, faster eye movements should result in larger error; however, reward has been shown to violate this trade-off (Manohar *et al.*, 2015). Overall, reward increased saccadic precision [reward × drug × group (3 × 2 × 2) repeated measures ANOVA: main effect of reward, *F*(2,94) = 3.795, *P = *0.026], but no other main effects or interactions were found (Fig. 5B). This reduction in saccadic error with increasing reward occurred regardless of group and in parallel with the previously mentioned increase in saccadic velocity in response to reward. Therefore, the observed reward-dependent velocity increases were not due to a speed/accuracy trade off. Thus, both saccadic velocity and precision improved with reward, as in previous work (Manohar *et al.*, 2015).

In this simple prosaccade task, reward magnitude had no effect on saccadic reaction time, in either group. Furthermore, there was no observed difference between the two groups in terms of reaction time reward sensitivity, and no effect of dopamine on reaction times. PD+ICD did, however, have faster overall reaction time [reward × drug × group (3 × 2 × 2) repeated measures ANOVA on reaction time: main effect of group, *F*(1,47) = 5.293, *P = *0.026], and reduced variability of reaction time [reward × drug × group (3 × 2 × 2) repeated measures ANOVA on standard deviation of reaction time: main effect of group, *F*(1,47) = 5.642, *P = *0.022] compared to PD-no-ICD.

### Traits predicting pupillary reward sensitivity

Muhammed *et al.* (2016) reported a significant negative correlation between pupil reward sensitivity and apathy scores, suggesting that in Parkinson’s disease reward sensitivity is modulated by apathy severity such that pupil reward sensitivity decreased with increasing apathy. In the current study, we performed a stepwise linear regression in the Parkinson’s disease population as a whole (*n *=* *49), beginning by including all LARS and BIS/BAS subcomponents as well as LEDD, DA-LEDD, cognitive impairment (MoCA), age, and symptom and disease duration as input variables. In the strongest model, mean pupil reward sensitivity (mean ON and OFF dopamine) correlated with two LARS subscales, action initiation (*S^r^* = 0.423, *P = *0.031) and intellectual curiosity (*S^r^* = −0.346, *P = *0.017) (but not LARS total), with greater intellectual curiosity and reduced action initiation correlating with increased reward sensitivity. Mean pupil reward sensitivity also correlated with DA-LEDD (*S^r^* = 0.284, *P = *0.031). A regression equation with these three input variables significantly predicted mean overall pupil reward sensitivity [*F*(3,45) = 5.616, *P = *0.002; *R*^2^ = 0.272].

### Traits predicting effect of dopamine on pupillary reward sensitivity

A second linear regression analysis was performed with the same predictor variables, this time with pupil reward sensitivity ON minus OFF as the dependent variable. In the whole Parkinson’s disease population, we found that BAS Fun-seeking (*β* = −0.456, *P < *0.001), BIS (*β* = −0.272, *P = *0.032), disease duration (*β* = −0.999, *P < *0.001) and symptom duration (*β  *=  0.728, *P = *0.033) scores were moderately predictive of the effect of dopamine on pupil reward sensitivity (*R*^2^ = 0.435, *P < *0.001). Thus, increased fun-seeking, behavioural inhibition, disease duration and decreased symptom duration was predictive of a decreased effect of dopamine on pupillary reward sensitivity. This is in line with our finding that the PD+ICD group had on average more severe Parkinson’s disease symptoms and greater behavioural impulsivity, as well as reduced effect of dopamine on pupil reward sensitivity. There was a trend towards a negative correlation indicating that with increasing overall Parkinson’s disease symptom severity (UPDRS total), patients exhibit decreasing effect of dopamine on reward sensitivity (*S^r^* = −0.264, *P = *0.066).

## Discussion

The findings presented here show that Parkinson’s disease patients with a history of ICDs have heightened sensitivity to exogenous monetary rewards cues both ON and OFF (overnight withdrawal) dopamine medication, as indexed by pupillary dilatation in response to reward cues. In contrast, PD-no-ICD patients show greater reward sensitivity ON medication compared to OFF (Fig. 3). Furthermore, because we were able to follow up many of our PD-no-ICD patients 4–5 years after they performed our eye tracking task, it was possible also to assess whether their original performance predicted development of subsequent impulsivity. The results suggest that pupil reward sensitivity is predictive of future development of ICDs in Parkinson’s disease (Fig. 4) and could therefore potentially provide a novel clinical measure to identify Parkinson’s disease patients at greater risk of developing ICDs. Although PD+ICD cases were not significantly more reward sensitive than PD-no-ICD patients in terms of motor response vigour, as indexed by mean peak saccade velocity, both groups were invigorated by reward cues. This effect of reward on response vigour could not be attributed simply to larger movements and was accompanied by increased saccadic precision (Fig. 5).

### The role of dopaminergic medications, including agonists, in impulse control disorders

The pupillary reward sensitivity findings were independent of total LEDD at the group level and did not significantly correlate with it across patients. However, there were some effects that suggest a potential impact of dopamine agonists. Across the patient groups, DA-LEDD was significantly correlated with pupil reward sensitivity when OFF dopaminergic medication and with respect to the mean of ON and OFF. But increased DA-LEDD did not predict increased proportional pupil change ON medication or crucially the difference between ON and OFF.

PD+ICD patients on agonists as a group compared to those on levodopa only had significantly greater pupil response to reward when OFF or with respect to the mean of ON and OFF dopaminergic medication. However, there was no significant difference between the groups when ON, nor crucially with respect to the effect of dopaminergic medication on reward sensitivity ON-OFF. Furthermore, when PD+ICD patients who were currently taking dopamine agonists were excluded and only those patients on only levodopa were compared to the PD-no-ICD cases, the main findings remain unchanged and significant (*cf.*Fig. 3). Taken together, these analyses demonstrate that the effects reported here regarding the difference in pupillary reward sensitivity between PD+ICD and PD-no-ICD cases in the ON and OFF states cannot be attributed solely to dopamine agonist use. Nevertheless, they show a potential modulatory effect of dopamine agonist dose that is not evident with total LEDD.

The results of many different studies have pointed to the possibility that ICDs in Parkinson’s disease might develop in response to dopamine agonist treatment interacting with underlying Parkinson’s disease pathophysiology and possibly personality traits (Weintraub *et al.*, 2013; Garcia-Ruiz *et al.*, 2014; Sharma *et al.*, 2015; Houeto *et al.*, 2016; Dawson *et al.*, 2018; Baig *et al.*, 2019). ICDs have been associated with the use of non-ergolinic oral dopamine agonists, such as rasagiline, ropinirole and pramipexole (Garcia-Ruiz *et al.*, 2014; Weintraub *et al.*, 2015), as well as ergoline-derivatives such as cabergoline (Weintraub *et al.*, 2010). However, there is no straightforward dose-dependent relationship across individuals (Ambermoon *et al.*, 2011; Corvol *et al.*, 2018; Dawson *et al.*, 2018), suggesting other factors are important for the overt manifestation of impulsive behaviour. These may include impaired learning from negative feedback, greater novelty seeking, enhanced delay discounting, rapid decision-making without sufficient evidence, reduced inhibitory control, and increased risk taking under conditions of ambiguity (for reviews see Basar *et al.*, 2010; Dalley *et al.*, 2011; Napier *et al.*, 2015; Voon *et al.*, 2017; Weintraub and Mamikonyan, 2019).

The findings reported here also suggest that the impulsive behaviours observed in PD+ICD patients might not be simply due to a direct effect of dopaminergic drug level on their sensitivity to reward. There was no difference in reward sensitivity between patient groups when ON dopamine medication, suggesting that impulse control disorders may not emerge simply because of a direct effect of dopaminergic drug level on reward sensitivity. It has previously been demonstrated that insensitivity to reward is an important factor in apathy in Parkinson’s disease and dopaminergic medication can improve motivation (Muhammed *et al.*, 2016). However, in the current study withdrawing dopaminergic medication did not reduce reward sensitivity in PD+ICD patients. This lack of differential effect of dopamine on reward sensitivity in the PD+ICD group might represent dopamine resistance or tolerance (Carriere *et al.*, 2014). Alternatively, it is possible that being OFF dopaminergic drugs for a longer period would show a reduction in reward sensitivity in this group, given dopamine agonists have a slightly longer pharmacokinetic half-life than levodopa (Rascol *et al.*, 1998).

It is also possible that the plateauing of reward sensitivity we observed here reflects the well-known U-shaped function of dopamine on brain functions (Gjedde *et al.*, 2010; Cools and D’Esposito, 2011; Vaillancourt *et al.*, 2013). Up to a certain point, increasing the level of dopamine in a brain region might boost reward sensitivity but then a threshold is met where further increases of dopamine have the effect of limiting or even reducing reward sensitivity. An optimal level of dopamine is considered a requirement for healthy reward evaluation and behavioural response. Saturation of the pupillary response to rewards might also account for the lack of increase in reward sensitivity ON medication in the PD+ICD group. Finally, there remains the possibility that some other factor, e.g. interactions between dopamine and other neurotransmitters such as serotonin or noradrenalin, might be crucial in influencing the motivational profile of PD+ICD patients (Cools, 2006; Dimatteo *et al.*, 2008; Campbell-Meiklejohn *et al.*, 2011; Cools and D’Esposito, 2011).

The data presented here would be consistent with the hypothesis that ICDs likely emerge in part as a consequence of dopamine replacement therapy, but on a background potentially of trait differences conferring susceptibility. In this regard, our finding that there were no significant differences in pupil reward sensitivity between PD+ICD patients who had currently active ICDs and those who had experienced ICD in the past would support such a view. Moreover, PD+ICD patients with active ICDs have significantly lower LEDD levels than PD+ICD patients without active ICD symptoms, but still had no difference in proportional pupil change reward sensitivity (ON versus OFF). These findings are in keeping with the proposal that ICD is likely to be a manifestation of an underlying phenotype conferring propensity to develop impulsive behaviours, which these patients share.

Proposed risk factors for the development of ICBs and ICDs include younger age and younger age at Parkinson’s disease onset and longer disease duration (Ceravolo *et al.*, 2009; Weintraub, 2009; Garcia-Ruiz *et al.*, 2014; Weintraub *et al.*, 2015; Antonini *et al.*, 2017). PD+ICD patients have also been reported to have higher levels of state and trait depression, aggressiveness and anxiety than PD-no-ICD patients. These individuals also have greater levels of trait impulsivity, choice impulsivity and novelty seeking (Isaias *et al.*, 2008; Voon *et al.*, 2010; Leroi *et al.*, 2012*a*,*b*; Vriend *et al.*, 2014; Houeto *et al.*, 2016). Consistent with previous studies, our PD+ICD cohort was found to be, on average, younger at diagnosis of Parkinson’s disease, with longer disease duration suggesting that they would have been taking dopaminergic medication for longer than the PD-no-ICD group. Furthermore, PD+ICD also had a greater load of motor symptoms (UPDRS Part 2) but no specific difference in depression (BDI), anhedonia (TEPS, SHAPS) or cognitive impairment (MoCA) compared to PD-no-ICD.

Regardless of which factors lead to ICD, the key findings presented here demonstrate that it is possible to show differences in reward sensitivity, indexed by pupillary response, between PD+ICD and PD-no-ICD cases (Fig. 3). Furthermore, such indices might be used to predict the likelihood of subsequent development of ICD (Fig. 4), although this requires replication and further validation.

### Reward processing in impulse control disorders

Previous work (Housden *et al.*, 2010; Voon *et al.*, 2010) has suggested that when cued with monetary rewards, PD+ICD patients have reduced tolerance for delayed gratification. In our study, patients had less time to evaluate the rewarding stimuli before a response was required. The subjective valuation and associated behavioural response reflect autonomic response to, and invigoration of reflexive responses by, reward cues. Therefore, the oculomotor behaviours we observed are likely to index incentive salience (Robinson and Berridge, 2001), or implicit sensitivity to rewarding stimuli, as opposed to conscious decision-making. From this perspective, heightened ‘wanting’ of rewards translates to heightened sensitivity for extrinsic rewards as well as a lower salience threshold for initiation of a reflexive behavioural response to obtain the reward. However, our findings suggest that the underlying incentive salience, or reward sensitivity, is not directly modulated by dopamine in PD+ICD because the autonomic pupil response to reward did not decrease when OFF dopamine. Additionally, the fact that pupillary reward sensitivity is predictive of future emergence of ICDs suggests that enhanced incentive salience of extrinsic rewards might even precede the behavioural disinhibition which characterizes pathological impulsive behaviour.

It has been previously reported that premorbid personality characteristics and traits might influence the nature and severity of ICDs (Houeto *et al.*, 2016). It is possible that the large variability in reward sensitivity within groups observed in the current study could be due to the wide range of ICD modalities reported by the PD+ICD group, leading to variable sensitivity to monetary rewards as opposed to other types of rewards. Monetary reward sensitivity may be higher in patients who suffer from money-related ICDs such as compulsive buying or gambling, and lower in patients with ICDs such as hypersexuality or compulsive eating. In this case, our monetary reward task would incentivize some patients to a greater degree than others, without accurately reflecting their underlying motivational disturbance.

### Comorbidity of impulsivity and apathy in Parkinson’s disease

In the current study, 78% of PD+ICD patients were either borderline or clinically apathetic, compared to 46% in the PD-no-ICD group. One possible helpful distinction in further understanding this issue might come from considering intrinsic versus extrinsic motivation. Behavioural apathy is characterized by a reduction in self-generated purposeful action in daily life: apathetic patients commonly struggle to initiate action of their own volition. Nevertheless, they are quite able to undertake actions if they are prompted to do so. Our oculomotor paradigm specifically utilized extrinsic reward stimuli to invigorate action (eye movements) in order to obtain the reward offered. Muhammed *et al.* (2016) reported that despite their reduced self-generated action, apathetic Parkinson’s disease patients were still incentivized and invigorated by extrinsic reward stimuli, as indexed by proportional pupil change and saccadic velocity, but they had reduced reward sensitivity compared to Parkinson’s disease patients without apathy. Similarly, in the current study, the majority of PD+ICD patients exhibited increased reward sensitivity for extrinsically presented reward cues while also suffering from behavioural apathy in their daily lives.

This suggests that these patients may be under-motivated when there are no immediate extrinsic reward cues, contributing to behavioural apathy, but over-motivated when responding to the presentation of, for instance, extrinsic monetary reward. Another possible interpretation of the counter-intuitive comorbidity of apathy and impulsivity is that these patients could experience heightened sensitivity for immediate pleasures, or associated reward cues while having difficulty generating or pursuing long term goals. Finally, it is worth noting that neurotransmitters other than dopamine, such as serotonin and noradrenaline, are known to be involved in arousal, incentive salience and reward processing (Korczyn and Keren, 1980; Guiard *et al.*, 2008; Manohar and Husain, 2015). Barber *et al*. (2018) recently provided evidence that atrophy of serotonergic neurons in the dorsal raphe nuclei correlates with apathy in REM (rapid eye movement) sleep behaviour disorder, a prodromal symptom of Parkinson’s disease, but found no correlations with dopamine.

### Limitations of the study

There were several limitations of this study. First, PD+ICD patients were recruited from a single tertiary centre from (unselected) referrals from several hospitals, so the patient groups might not be representative of typical cases. Although we report the data from 49 Parkinson’s disease cases ON and OFF medications, the sample sizes are relatively small. The PD+ICD group contained individuals with current and previous ICDs and was therefore potentially heterogeneous and there were baseline factors that might also have been potential confounding factors, although we have tried to control for all these issues in the analyses performed herein.

## Conclusion

This study demonstrates that PD+ICD patients have heightened sensitivity to exogenous monetary reward cues both ON and OFF (overnight withdrawal) dopamine medication, whereas PD-no-ICD patients show increased reward sensitivity ON medication compared to OFF. Although the effects are not accounted for by total LEDD, there is an impact of DA-LEDD on pupil reward sensitivity, but this cannot account for the entire pattern of results. Moreover, the difference between PD+ICD and PD-no-ICD groups remains significant even when PD+ICD cases on dopamine agonists are excluded. The findings support the view that ICDs emerge in Parkinson’s disease in response to dopamine replacement therapy and that hypersensitivity to rewards in some susceptible individuals might contribute to impulsive behaviours in Parkinson’s disease. However, the findings argue against a simple relationship between dopamine level and reward sensitivity. Many PD+ICD patients were also found to experience behavioural apathy and impulsivity comorbidly, suggesting that aberrant reward sensitivity is just one component of a dysfunctional system which may incorporate functional changes in other neurotransmitter systems. The simple oculomotor paradigm employed here also provides the potential for novel clinical measurement of impulsivity and could be used to identify patients who are at greater risk to develop ICDs in the future.

## Funding

This work was supported by a Wellcome Trust Principal Research Fellowship to MH, the National Institute for Health Research Oxford Biomedical Research Centre, a Wellcome Trust Clinical Research Training Fellowship to KM and a Medical Research Council Clinician Scientist Fellowship MR/P00878X to SM.

## Competing interests

The authors report no competing interests.

## Supplementary Material

awaa198_Supplementary_Data

## Abbreviations

DA-LEDD =: dopamine agonist levodopa equivalent daily dose
ICB = impulse control behaviour; ICD =: impulse control disorder
LARS =: Lille Apathy Rating Scale
PD+ICD =: Parkinson’s disease with ICD
PD-no-ICD =: Parkinson’s disease without ICD
QUIP =: Questionnaire for Impulsive-Compulsive Disorders in Parkinson’s Disease
UPDRS =: Unified Parkinson’s Disease Rating Scale
