# Supplementary material for: Dopamine and reward hypersensitivity in Parkinson’s disease with impulse control disorder
Source: Brain. 2020 Aug 6;143(8):2502–18. doi: 10.1093/brain/awaa198 (PMC7447523; doi:10.1093/brain/awaa198)
Supplement: awaa198_Supplementary_Data [file awaa198_supplementary_data.zip › awaa198-suppl_data/brain-2019-01617-File008.pdf]

Hypersensitivity to rewards in PD+ICD: Supplementary Material

Abbreviations:
PPC: Proportional pupil change
RS: Reward sensitivity
LEDD: levodopa equivalent daily dose
DA-LEDD: dopamine agonist levodopa equivalent daily dose

ICD TYPE	PD+ICD anytime	PD+ICD current	PD-no-ICD anytime	PD-no-ICD current (4-5 years after behavioural testing)	Total Anytime	Total Current
**Pathological Gambling**	4	3	0	0	4	3
**Compulsive Sexual Behaviour**	8	6	6	5	14	11
**Compulsive Buying**	14	8	7	4	21	12
**Compulsive Eating**	12	11	6	6	18	17
**Hobbyism / punding**	14	10	10	6	24	16
**DDS**	2	n/a	0	n/a	2	n/a
**Multiple ICD Modalities**	16	10	10	6	26	16
N	23	23	24	24	47	47

Supplementary Table 1. Frequencies of ICD subtypes

Frequencies of different ICD subtypes reported by patients using the QUIP anytime and current (Questionnaire for impulsive-compulsive disorders in Parkinson's disease). DDS represents Dopamine Dysregulation Syndrome. Multiple ICD modalities refer to patients who reported the presence of more than one concomitant ICD type. For PD+ICD 'anytime' score represents the presence of ICD anytime in ones' life for a period of at least 4 weeks and 'current' score represents the presence of ICD for a period of at least 4 weeks at the time of the QUIP and behavioural data collection. At the time of behavioural testing, no-one in the PD-no-ICD group had any self-reported or clinically identified ICDs. In PD-no-ICD the 'anytime' score represents the presence of ICD for a period of at least 4 weeks since behavioural testing, approximately 4 – 5 years earlier. PD-no-ICD current score represents the presence of ICD for a period of at least 4 weeks at the time of QUIP collection approximately 4 – 5 years after behavioural testing.

Dopaminergic and Cholinergic medication in PD+ICD:

PD+ICD Patient	PD Medication at time of testing	Levodopa or agonists	Levodopa Equivalent Dose (mg/24hrs)	Dopamine Agonist Equivalent Dose (mg/24hrs)	Previous Agonist Use (max dose)	Previous Agonist Withdrawal (Years before testing)	Current Cholinergic Medication Use
**1**	Ropinirole, Sinemet	Both	1140	240	Ropinirole (20mg)	No	No
**2**	Levodopa, Rotigotine (neupro patch)	Both	982	182	Ropinirole (24mg)	No	No
**3**	Sinemet	Levodopa	400	0	Ropinirole (12mg)	Yes (?)	No
**4**	Sinemet, Ropinirole	Both	540	240	Ropinirole (20mg)	No	No
**5**	Sinemet, Rasagiline, Rivastigmine	Levodopa	850	0	Ropinirole (10mg)	Yes (?)	Yes – Rivastigmine (6mg)
**6**	Sinemet, Ropinirole	Both	560	160	Ropinirole (14mg)	No	No
**7**	Sinemet (Co-careldopa), Rasagiline	Levodopa	250	0	No	n/a	No
**8**	Sinemet, Rasagiline, Ropinirole	Levodopa	520	120	Ropinirole (9mg)	No	No
**9**	Madopar	Levodopa	750	0	Ropinirole (4mg)	Yes (?)	No
**10**	Sinemet, Madopar, Rotigotine (neupro patch)	Both	742	242	Ropinirole (14mg)	No	No
**11**	Madopar, Entacapone	Levodopa	133	0	Pramipexole (3.15mg)	Yes (?)	No
**12**	Co-Beneldopa, Ropinirole	Both	640	240	Ropinirole (24mg)	No	No
**13**	Sinemet, Rasagiline, Ropinirole	both	590	240	Ropinirole (12mg)	No	No
**14**	Stalevo, Rasagiline	Levodopa	599	0	Ropinirole (8mg)	Yes (?)	No
**15**	Stalevo, APO-Go Pen (Apomorphine),	Both	1108	542	Rotigotine (12mg)	No	No

	Rasagiline, Rotigotine (neupro patch), DBS						
**16**	Sastravi, Co-Beneldopa, Rasagiline, Amantadine, Entacapone	Levodopa	919	120	Ropinirole (12mg)	No	No
**17**	Stalevo, Sinemet, Donepezil	Levodopa	836	0	No	n/a	Yes – Donepezil
**18**	Stalevo, DBS	Levodopa	1197	0	Pramipexole (3.15mg)	Yes (2.5)	No
**19**	Madopar, Co-Beneldopa, Rasagiline, Amantadine, Entacapone	Levodopa	865	0	Pramipexole (3.15mg)	Yes (6)	No
**20**	Madopar, Entacapone	Levodopa	1480	0	Ropinirole (24mg)	Yes (2.5)	No
**21**	Amantadine, Ropinirole, Madopar, Rasagiline	Both	575	440	Ropinirole (24mg)	No – reduced dose (6)	No
**22**	Stalevo, Amantadine	Levodopa	400	0	Pramipexole (?)	No (10)	No
**23**	Pramipexole, Madopar	Both	600	150	Pramipexole (1.05mg)	n/a	
**Mean**	n/a	n/a	716	127	n/a	Yes (n=9) (mean: 5.4 years (n=5))	Yes (n=2)

Supplementary Table 2: PD+ICD medication types: Levodopa vs dopamine agonists

Dopaminergic and Cholinergic medication in PD-no-ICD:

PD-no-ICD Patient	PD Medication at time of testing	Levodopa or agonists	Levodopa Equivalent Dose (mg/24hrs)	Dopamine Agonist Equivalent Dose (mg/24hrs)	Previous Agonist Use	Agonist Withdrawal (Years since)	Current Cholinergic Medication Use
**1**	Sinemet	Levodopa	600	0	No	n/a	No
**2**	Sinemet, Pramipexole	Both	204	13	Yes	n/a	No
**3**	Ropinirole, Madopar	Both	575	200	Yes	n/a	No
**4**	Stalevo, Amantadine, Pramipexole, Selegiline	Both	852	78	Yes	n/a	No
**5**	Madopar, Rotigotine, Rasagiline	Both	410	243	Yes	n/a	No
**6**	Sinemet, Entacapone, Pramipexole	Both	1156	485	Yes	n/a	No
**7**	Sinemet	Levodopa	600	0	No	n/a	No
**8**	Ropinirole, Co-beneldopa	Levodopa	555	480	Yes	n/a	No
**9**	Rasagiline, Co-beneldopa, Gabapentin	Levodopa	250	0	Yes	n/a	No
**10**	Sinemet, Rasagiline	Levodopa	1050	0	No	n/a	No
**11**	Co-beneldopa	Levodopa	300	0	No	n/a	No
**12**	Ropinirole, Madopar	Both	223	120	Yes	n/a	No
**13**	Sinemet	Levodopa	150	0	No	n/a	No
**14**	Co-beneldopa	Levodopa	300	0	No	n/a	No
**15**	Pramipexole, Madopar	Both	134	38	Yes	n/a	No
**16**	Rasagiline, Pramipexole, Madopar	Both	202	74	Couldn’t access clinical letters	n/a	No

**17**	Sinemet, Pramipexole	Both	400	143	Yes	n/a	No
**18**	Madopar	Levodopa	150	0	No	n/a	No
**19**	Sinemet, Trihexyphenidyl	Levodopa	563	0	Couldn't access clinical letters	n/a	Yes - Trihexyphenidyl 2,5mg
**20**	Madopar, Ropinirole	Both	1250	100	Yes	n/a	No
**21**	Co-careldopa	Levodopa	150	0	No	n/a	No
**22**	Rasagiline, Stalevo	Levodopa	850	0	No	n/a	No
**23**	Co-beneldopa, Amantadine	Levodopa	500	0	No	n/a	No
**24**	Co-careldopa, Selegiline, Amantadine	Levodopa	850	0	Couldn't access clinical letters	n/a	No
**25**	Pramipexole	Agonist	53	0	Yes	n/a	No
**26**	Stalevo	Levodopa	600	0	No	n/a	No
**Mean**	n/a	n/a	497	76	Yes (n=11)<br>No (n=10)<br>Unknown (n=5)	n/a	Yes (n=1)

Supplementary Table 3: PD-no-ICD medication types: Levodopa vs dopamine agonists

PD+ICD	Compulsive Gambling		Compulsive Sexual behaviour		Compulsive Buying		Compulsive Eating		Hobbyism / Punding		DDS	Total modalities Current	Total modalities Anytime	Anytime minus Current
	Current	Anytime	Current	Anytime	Current	Anytime	Current	Anytime	Current	Anytime	Anytime			
**1**	0	0	0	0	0	0	1	1	0	0	0	1	1	0
**2**	0	0	1	1	0	0	0	0	1	1	0	2	2	0
**3**	0	0	0	0	0	1	0	1	0	1	0	0	3	3
**4**	0	0	0	0	0	0	0	0	0	1	0	0	1	1
**5**	0	0	0	0	1	1	1	1	1	1	0	3	3	0
**6**	0	0	0	0	0	0	1	1	0	0	1	1	2	1
**7**	0	0	1	0	0	0	1	1	1	1	1	3	3	0
**8**	0	0	1	1	1	1	1	1	1	1	1	4	5	1
**9**	0	0	0	0	1	1	1	1	1	1	1	3	4	1
**10**	0	0	0	0	1	1	0	0	1	1	0	2	2	0
**11**	1	1	0	0	0	0	1	1	1	1	0	3	3	0
**12**	1	1	1	1	1	1	0	0	1	1	0	4	4	0
**13**	0	0	0	0	1	1	1	1	0	0	0	2	2	0
**14**	0	0	1	1	0	0	0	0	0	0	1	1	2	1
**15**	0	0	0	0	0	1	0	0	0	0	0	0	1	1
**16**	0	0	0	0	0	1	1	1	0	1	0	1	3	2
**17**	0	0	0	0	0	1	0	0	0	0	0	0	1	1
**18**	0	0	0	1	0	1	0	0	0	0	1	0	3	3
**19**	0	1	0	0	0	1	0	0	0	1	0	0	3	3
**20**	0	0	0	1	0	0	0	0	0	0	0	0	1	1
**21**	0	0	0	0	1	1	1	1	1	1	0	3	3	0
**22**	1	1	1	1	1	1	1	1	1	1	0	5	5	0
**23**	0	0	1	1	0	0	0	0	0	0	0	1	1	0
**TOTAL**	**3**	**4**	**7**	**8**	**8**	**14**	**11**	**12**	**10**	**14**	**6**	**39**	**58**	**19**

Supplementary Table 4: Covariance matrix for ICD types in PD+ICD. DDS = dopamine dysregulation syndrome. Anytime refers to the QUIP-anytime questionnaire which identifies periods of ICD at any time in a Parkinson's disease patient's life that lasted for at least 4 weeks. Current refers to the QUIP-current questionnaire which identifies current ICDs in a Parkinson's disease patient that lasted for at least 4 weeks.

PD-no-ICD	Compulsive Gambling		Compulsive Sexual behaviour		Compulsive Buying		Compulsive Eating		Hobbyism / Punding		DDS	Total modalities Current	Total modalities Anytime	Anytime minus Current
	Current	Anytime	Current	Anytime	Current	Anytime	Current	Anytime	Current	Anytime	Anytime			
1	0	0	0	0	0	0	0	0	0	0	0	0	0	0
2	0	0	1	1	0	0	1	1	0	0	0	2	2	0
3	0	0	0	0	0	0	0	0	0	0	0	0	0	0
4	0	0	0	0	0	0	0	0	1	1	0	1	1	0
5	0	0	0	0	0	0	0	0	0	1	0	0	1	1
6	0	0	0	0	0	0	0	0	0	0	0	0	0	0
7	0	0	0	0	1	1	1	1	0	0	0	2	2	0
8	0	0	0	1	0	1	0	1	0	1	0	0	4	4
9	0	0	0	0	0	0	0	0	0	0	0	0	0	0
10	0	0	0	0	0	0	0	0	0	0	0	0	0	0
11	0	0	0	0	0	0	0	0	0	0	0	0	0	0
12	0	0	1	1	0	0	0	0	0	0	0	1	1	0
13	n/a	n/a	n/a	n/a	n/a	n/a	n/a	n/a	n/a	n/a	n/a	n/a	n/a	n/a
14	0	0	0	0	0	0	0	0	0	0	0	0	0	0
15	0	0	0	0	0	0	0	0	0	0	0	0	0	0
16	0	0	0	0	0	0	0	0	0	1	0	0	1	1
17	0	0	0	0	0	1	0	0	1	1	0	1	2	1
18	n/a	n/a	n/a	n/a	n/a	n/a	n/a	n/a	n/a	n/a	n/a	n/a	n/a	n/a
19	0	0	1	1	0	0	0	0	0	0	0	1	1	0
20	n/a	n/a	n/a	n/a	n/a	n/a	n/a	n/a	n/a	n/a	n/a	0	n/a	n/a
21	0	0	0	0	0	1	0	0	0	1	0	0	2	2
22	0	0	0	0	0	0	0	0	0	0	0	0	0	0
23	0	0	0	0	0	0	0	0	0	0	0	0	0	0
24	0	0	1	1	1	1	1	1	1	1	0	4	4	0
25	0	0	0	0	0	0	0	0	1	1	0	1	1	0
26	n/a	n/a	n/a	n/a	n/a	n/a	n/a	n/a	n/a	n/a	n/a	n/a	n/a	n/a
**TOTAL**	**0**	**0**	**4**	**5**	**2**	**5**	**3**	**4**	**4**	**8**	**0**	**13**	**22**	**9**

Supplementary Table 5: Covariance matrix for ICD types in PD-no-ICD developed in the 4-5 years since behavioural testing. DDS = dopamine dysregulation syndrome. Anytime refers to the QUIP-anytime questionnaire which identifies periods of ICD at any time in a Parkinson's disease patient's life that lasted for at least 4 weeks. Current refers to the QUIP-current questionnaire which identifies current ICDs in a Parkinson's disease patient that lasted for at least 4 weeks.

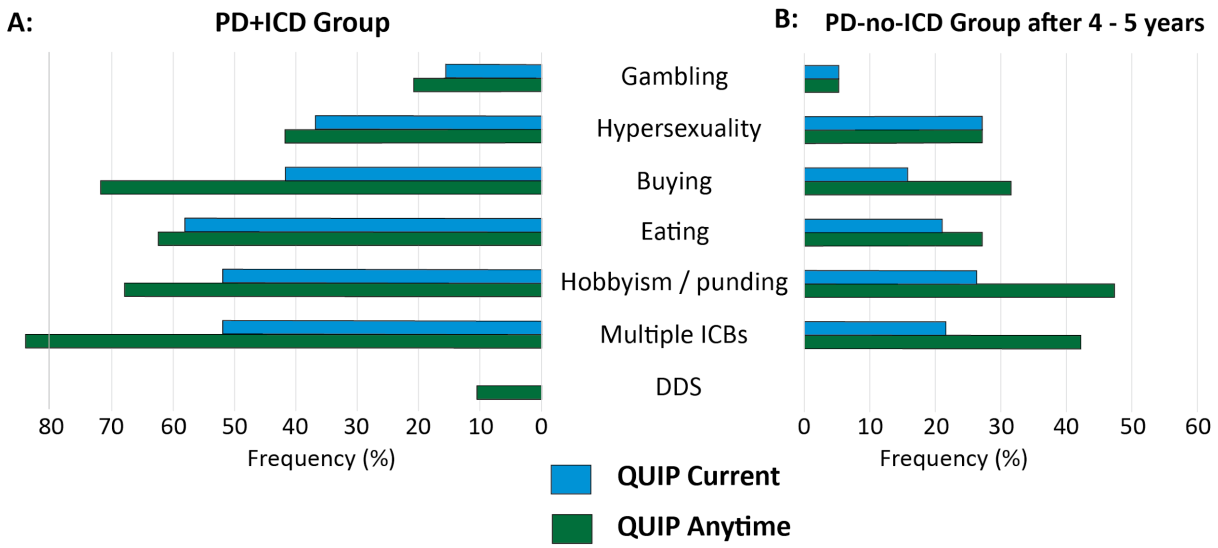

Supplementary Figure 1: Frequencies of ICD subtypes

(A) Frequencies of ICD subtypes in PD+ICD (left) currently (at time of behavioural testing) (blue) and anytime in their life for a period of at least 4 weeks (green) as a percentage. Current ICD frequencies are reduced because of changes made to the patient's medication regime to account for the presence of ICDs. **(B)** Frequencies of ICD subtypes in the PD-no-ICD group (right): At the time of behavioural testing PD-no-ICD had no self-reported or clinically identified ICDs. However, 4 – 5 years later (when behavioural testing of the PD+ICD group was conducted), ICD frequency data was collected from the PD-no-ICD group, therefore their frequencies represent ICDs which emerged after the behavioural testing took place. PD-no-ICD anytime (green) frequencies represent the presence of ICDs for a period of at least 4 weeks in the 4-5 years since behavioural testing. These frequencies are greater than the 4-5 years later (current) frequencies (blue) because of changes made to the patient's medication regime to account for the presence of ICDs. DDS: dopamine dysregulation syndrome.

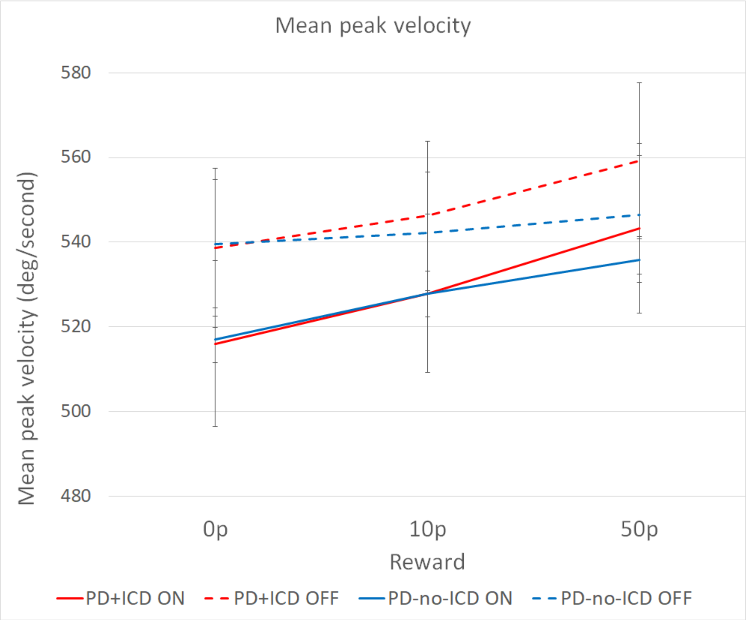

Supplementary Figure 2: Mean peak saccadic velocity as a function of reward level in PD+ICD and PD-no-ICD ON and OFF dopamine.

There were no significant differences between groups in mean peak saccadic velocity and both groups had greater velocities OFF dopamine compared to ON dopamine. Both PD+ICD and PD-no-ICD had increased invigoration of saccades for larger rewards. Across groups there was greater saccadic reward sensitivity ON dopamine. There was a non-significant trend towards greater overall invigoration by reward in PD+ICD, driven by reduced reward sensitivity OFF dopamine in PD-no-ICD. Error bars represent standard error of the mean.

Supplementary Analyses

1. Post hoc analyses: PD+ICD vs PD-no-ICD

1.1 2 (drug) repeated measure ANOVA in each group on pupil change reward sensitivity values

We calculated the reward sensitivity in each drug condition and considered each group separately. In the PD-no-ICD group, pupil reward sensitivity was lower when OFF dopamine than ON [paired two-samples t-test on reward sensitivity, $F(1,25)=7.866$, $P=0.010$] (***Figure 3a***), whereas for the PD+ICD group, drug made no significant difference. Comparing the groups directly, when OFF dopamine, PD-no-ICD were less reward sensitive than PD+ICD [unpaired t-test on reward sensitivity, $F(1,47)=7.990$, $P=0.007$], as shown by their shallower slope (***Figure 3b,*** *dotted lines*), whereas when ON dopamine, there was no significant group difference. Thus, the two groups showed differential effects of dopamine on reward sensitivity.

Unpaired t-test on mean reward sensitivity across drug condition (RS ON + RS OFF) found no difference between PD+ICD and PD-no-ICD. Therefore, overall PD+ICD do not have significantly greater reward sensitivity *per se*, but rather, they lack dopaminergic modulation of their pupillary reward response, with heightened reward sensitivity OFF medication, which might contribute to the emergence of ICBs in this group.

1.2 2 (drug) x 2 (group) repeated measure ANOVA on Residual Velocity Reward Sensitivity values

Similar to the pupil analysis, to break down the interaction, we calculated a reward sensitivity statistic on the velocity residuals. Overall, residual velocity reward sensitivity was greater ON dopamine (Drug x Group (2x2) 2-way repeated measures ANOVA on reward sensitivity, main effect of Drug [$F(1,47)=4.381$, $P=0.042$]) but with no significant difference between the groups in reward sensitivity as indexed by mean peak residual velocity. No interaction was found between drug and group.
Decomposing these results further, while there was no difference between PD+ICD residual velocity reward sensitivity ON and OFF dopamine there was a difference in PD-no-ICD with greater reward sensitivity ON than OFF dopamine [2-sample paired t-test, reward sensitivity ON vs. OFF dopamine in PD-no-ICD, $F(1,25)=5.178$, $P=0.032$] (*Figure* 5a).

1.3 Are PD+ICD undermotivated for no-reward (0p) condition?

We tried to assess whether the steeper reward sensitivity slope in PD+ICD was driven by reduced pupillometric and behavioural response to the no-reward 0p condition. PD+ICD patient's pupils did dilate less than PD-no-ICD in response to the 0p reward stimuli [Drug x Group (2x2) repeated measures ANOVA, main between-subjects effect of Group, $F(1,47)=13.778$, $P<0.001$], however this could be because overall PD+ICD had smaller proportional pupil responses due to larger baseline pupil sizes and thus less scope for further dilation. PD+ICD also had reduced pupil change in response to the 10p and 50p reward condition despite their steeper reward sensitivity slopes. Nevertheless, this reduced response to 0p could reflect reduced motivation for action without extrinsic incentivisation.

To look at the relationship between apathy and impulsivity in PD+ICD further we performed a one-way ANOVA analysis with apathy group (no apathy (n=7) , apathy (n=16) as a between subjects variable (2-Group x 2-Drug RM ANOVA on proportional pupillary change in 0p condition). There was no between subjects effect of Apathy Group on proportional pupil change to 0p although there was a main effect of Drug [$F(1,21) = 5.821$, $P=0.025$], with greater pupil change OFF dopamine, and a trend towards an Apathy Group x Drug interaction [$F(1,21) = 2.924$, $P = 0.102$].

This indicates that PD+ICD with apathy did not have reduced autonomic pupil response to 0p than PD+ICD without apathy. Furthermore, no group effect was found in terms of residual saccadic velocity or variability, indices of task performance.

Additionally, Spearman's correlations revealed that LARS total positively correlated with pupil change to 0p ON dopamine in PD+ICD [$S^r=0.509$, $P=0.013$] indicating greater pupil response to 0p with greater apathy PD+ICD ON dopamine.

1.4 Correlations between daily dose (LEDD and DA-LEDD) and apathy (LARS) in 2 patient groups (PD+ICD (n=23) and PD-no-ICD (n=26)).

The PD+ICD patients in our study had significantly higher mean levodopa equivalent daily dose than the PD patients without impulsivity. However, we found no correlation across the whole sample of Parkinson's patient between apathy (LARS Total) and daily dopaminergic dose (LEDD / DA-LEDD) and only one significant LARS subscale correlation – between the LARS self-awareness component and DA-LEDD [$S^r=-0.340$, $P=0.017$]. To investigate whether there was any relationship in the PD+ICD and PD-no-ICD groups we performed a post hoc correlation analysis. The only significant correlation we found was between LARS self-awareness and DA-LEDD in the PD+ICD group [$S^r=-0.518$, $P=0.011$]; increased DA-LEDD dose predicts reduced self-awareness. This was not a significant correlation in the PD-no-ICD alone, suggesting that the effect in

the whole population was driven by the Parkinson's patients with impulsivity. Nevertheless, the main finding here is that there was no relationship between level of dopamine dose and overall apathy level in either PD+ICD or PD-no-ICD or in the whole population.

1.5 Correlations between daily dose (LEDD and DA-LEDD) and mean peak proportional pupil change in 0p (no-reward) condition ON and OFF Dopamine.

In order to investigate further whether apathy may relate to dopamine level, we performed correlations between daily dopamine dose (LEDD / DA-LEDD) and proportional pupil change in the 0p (no-reward) condition. The 0p condition could potentially act as an index of intrinsic, self-generated motivation since in this condition there is no extrinsic reward stimulus, yet the subject is still required to respond to the visual stimulus by completing a saccade to the peripheral target. However, we found no significant correlations in either group between levodopa equivalent dose or dopamine agonist equivalent dose ON or OFF dopamine showing that in this sample there is no relationship between dopamine dose and pupil response to the no-reward condition, thus no evidence that dopamine dose relates to intrinsic motivation in these patients.

1.6 Correlations between daily dose (LEDD / DA-LEDD) and change in UPDRS motor examination (UPDRS 3 OFF minus ON).

The Parkinson's disease patients with impulsivity in our group also had greater overall PD symptom severity (UPDRS total) as well as significantly greater scores on the subscales UPDRS part 1, 2, 4 and in disease stage (Hoehn and Yahr score), as reported in figure (1). Interestingly there was no difference between the two groups in UPDRS part 3, the motor examination ON or OFF, or OFF minus ON (a metric for the efficacy of medication on motor symptoms). We performed a correlation analysis to look at the relationship between daily dose (LEDD / DA-LEDD) and the effect that medication has on the patient's motor symptoms (UPDRS OFF minus ON) in each group (PD+ICD and PD-no-ICD). We found no relationship between DA-LEDD, daily dopamine agonist dose, and UPDRS OFF-ON in either group, however we did find significant correlations between LEDD, daily overall dose, and UPDRS OFF-ON in PD+ICD [S^r=0.524, P=0.010] and PD-no-ICD [S^r=0.479, P=0.013]. This indicates, somewhat unsurprisingly, that patients on more medication had a stronger drug effect on their motor symptoms.

1.7 Covariate analysis – Possible confounds of baseline characteristics on proportional pupil change

We performed a series of covariate analyses to investigate whether differences in baseline characteristics account for the between-group differences that we report. We performed additional (3 Reward x 2 Drug x 2 Group) ANOVAs with baseline characteristics (age, age at diagnosis, disease duration, symptom duration, UPDRS 1, 2, 4, Total, LEDD, LARS Total, MOCA) as a covariate on proportional pupil change (PPC). Our effects remained even when age at diagnosis and disease duration were included as factors.

The Reward x Drug x Group 3-way interaction was lost when UPDRS 1, 2 and Total were added as covariates on proportional pupil change. However, there were no significant main effects between subjects on UPDRS 1, 2, 3 (ON or OFF), 4 or Total UPDRS. A between subjects main effect of DA-LEDD was found when added as a covariate to PPC [$F(1,46)=5.481$, $P = 0.024$].

The only significant correlation between UPDRS Total or subscales and PPC reward sensitivity (RS) ON, OFF, mean across drug condition, and ON-OFF is between UPDRS Part 2 and PPC RS ON-OFF [S^r=-.359, P=0.011]. **This correlation shows that increased self-reported motor symptom load is associated with a reduced effect of dopamine on pupillary reward sensitivity.**

These results suggest that, in general, the difference between groups is unlikely to be related to other clinical dimensions, apart from the fact that increased self-reported motor symptomatology (one possible marker of disease severity) is associated with a more blunted pupillary response to reward *in the comparison between ON* and *OFF state.*

1.8 Could the fact that some patients were taking anticholinergic medication have confounded our pupillometric findings?

Cholinergic medications are commonly prescribed to Parkinson's disease patients and are known to have direct mydriatic effects on the pupil. In the PD+ICD group two patients were taking cholinergic medication (rivastigmine n=1, donepezil n=1). In the PD-no-ICD group, only one patient was currently taking cholinergic medication (trihexyphenidyl). Since so few patients were taking these medications it is unlikely to have affected the overall results, however, to check whether this factor may have confounded our pupil reward sensitivity findings we reanalysed the data after excluding all patients who were currently taking cholinergic medication and the overall findings were not affected (3 Reward x 2 Drug x 2 Group RM ANOVA on proportional pupil change).

After excluding these patients we still found a main effect of Reward [$F(2,88)=22.333$, $P=<0.001$], and Drug [$F(1,44) = 12.702$, $P=0.001$], as well as a between subjects main effect of Group [$F(1,44)=8.705$, $P=0.005$], and importantly the 3 way Reward x Drug x Group interaction was also preserved in this analysis [$F(1,44)=8.705$, $P=0.005$]. There was also a trend towards a Reward x Group interaction [$F(2,88) = 2.905$, $P<0.060$].

1.9 Duration of the prodrome period is associated with an increased effect of dopamine on pupil reward sensitivity:

We calculated a new variable - Prodrome time – by subtracting disease duration (years since diagnosis) from symptom duration (years since symptoms started) which represents the duration of time that patients were symptomatic before they were diagnosed with Parkinson's disease.

We found that prodrome time positively correlates with the effect of dopamine medication on pupil reward sensitivity (pupil RS ON dopamine minus OFF dopamine) [$Sr=0.345$, $P=0.020$]. People with longer prodrome periods are more likely to have larger effects of dopamine on pupillary reward sensitivity. Furthermore, our post hoc correlation analysis showed that Parkinson's patients with and without ICD on overall more medication (LEDD), but not specifically more agonists (DA-LEDD) had a stronger drug effect on their motor symptoms (UPDRS 3 OFF-ON) (supplementary analysis 1.6).

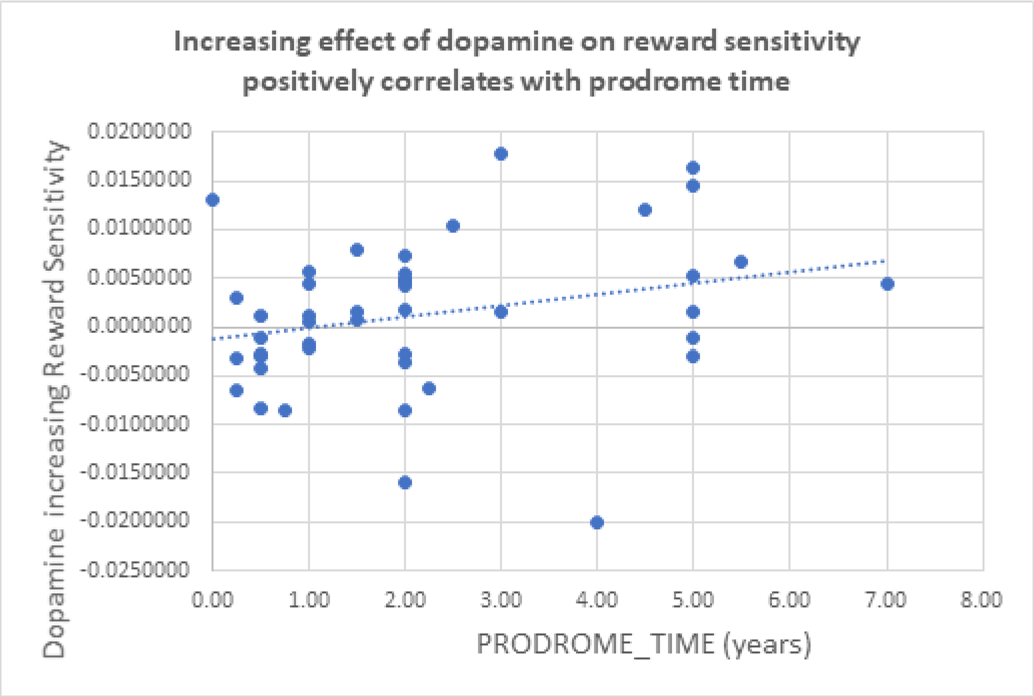

1.10 Do PD+ICD and PD-no-ICD display differential learning effects?

Learning differences have previously been demonstrated between Parkinson's patients with and without impulsivity in other research reports. To examine any effects of learning we measured saccadic reaction times for early and late blocks. There was no effect of learning, and no modulation by drug or group (Drug x Group x Reward x early/late RM ANOVA). Thus, both groups showed similar learning. Secondly, because each patient was tested on two days, in a randomised order, we also included a factor of "session" (first / second) into the drug analysis, performing a 4-Way 2 time courses (Early/Late) x 3 Reward x 2 Drug x 2 Group repeated measures ANOVA on saccadic reaction times. This analysis revealed no main effect of Drug, Reward, or between subjects effect of Group, no main effect of time course and no significant interactions. Thus, there was no overall evidence for differences in learning being a confounding factor.

1.11 Correlation matrix of impulsivity and apathy questionnaire variables

78% of the Parkinson's disease patients with impulsivity in this study also had co-occurring apathy. Furthermore, 46% of patients without impulsivity were also apathetic at the time of testing, and 60% of these patients went on to develop behavioural impulsivity in the 5 years following this. We were interested to look at the relationship between apathy and impulsivity in these patients so we performed a correlation analysis with all of the impulsivity and apathy measures that we collected. Due to the large number of correlations in this Spearman's correlation matrix we also performed Bonferroni corrections for multiple comparisons.

After performing robust Bonferroni corrections for multiple comparisons for our analysis of how impulsivity and apathy relate, only two significant correlations remained for the whole population: between BAS-drive and UPPS-P total [S^r=0.485, P<.001] - increasing drive correlates with increasing underlying impulsive traits and between BIS and UPPS-P [S^r=-0.569, P<0.001] - increasing behavioural inhibition correlates with decreasing underlying impulsive traits:

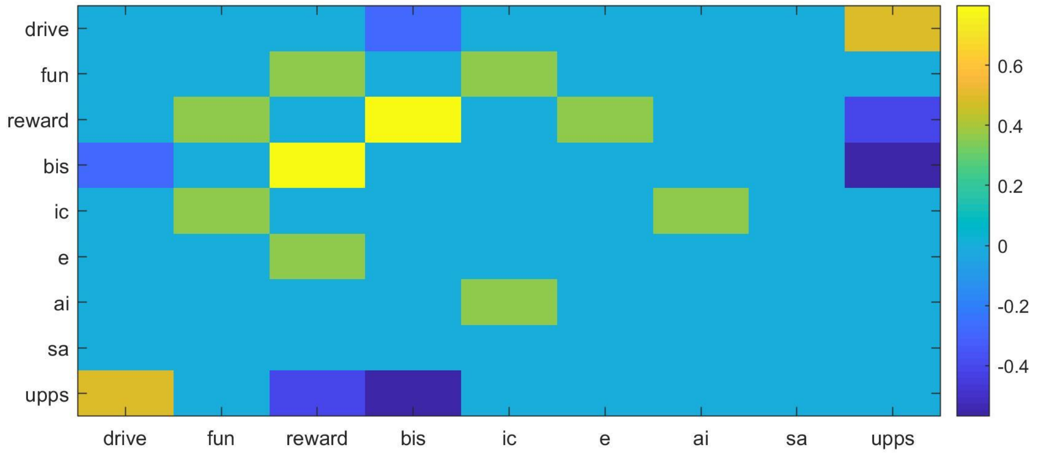

Drive = BAS-drive; fun = BAS-fun seeking; reward = BAS-reward responsivity; bis = Behavioural inhibition system; ic = LARS – intellectual curiosity; e = LARS-emotion; ai = LARS- action initiation; sa = LARS-Self awareness; upps-p = Urgency, Premeditation, Perseverance, Sensation Seeking, Positive Urgency scale.

1.12 Within-Group Correlations

Considering that there are differences in baseline characteristics (such as PD severity) but few significant correlations across the whole population of PD patients, we conducted a series of correlations in each group to assess within-group effects.

Firstly, we wanted to look at whether PD symptom severity correlated with patients' reward-related pupillary response. In PD+ICD there were no significant correlations between PD severity (UPDRS total or subscales) with proportional pupil change reward sensitivity ON, OFF, overall (mean for ON and OFF) or the effect of dopamine on reward sensitivity (ON-OFF) except between UPDRS Part 4 (motor complications) and proportional pupil change reward sensitivity OFF dopamine: [S^r=0.489, P=0.011] indicating that increased motor complications (dyskinesia and/or OFF states) predicts greater pupillary reward sensitivity OFF dopamine, but not ON dopamine.

Next, we wanted to investigate the relationship between impulsivity (UPPS-P and BISBAS scales) and pupil reward sensitivity in each group separately. In PD+ICD patients, we found significant correlations between proportional pupil change RS ON-OFF and BAS-Fun seeking [S^r=-0.487, P=0.018] and BAS-Reward responsiveness [S^r=-0.438, P=0.036]. In PD+ICD increased (self-reported) reward responsiveness and fun seeking is associated with a decreased effect of dopamine on reward sensitivity. This suggests these behaviours or traits facilitate the resistance of reward sensitivity (as indexed by pupillary dilation to reward) to overnight dopamine withdrawal. We found no significant correlations between the UPPS-P questionnaire and any pupil reward sensitivity measure.

In the PD-no-ICD group we also found a significant correlation between PPC RS ON-OFF and BAS-Fun seeking [S^r=-0.416, P=0.035], but also between BAS-Fun seeking and PPC RS ON dopamine [S^r=-0.507, P=0.008] and mean overall PPC RS [S^r=-0.403, P=0.041], but not PPC RS OFF dopamine. Therefore, in PD patients without impulsivity increased fun-seeking behaviour is also associated with a decreased effect of dopamine on reward sensitivity, but also with decreased mean overall pupil reward sensitivity

driven by reduced reward sensitivity ON dopamine. We found no significant correlations between the UPPS-P questionnaire and any pupil reward sensitivity measure in PD-no-ICD.

Finally, we performed correlations to look at the relationship between apathy (LARS total and subscales) and proportional pupil change RS in both groups separately. In PD patients with ICD, increased apathy (LARS total) predicts a reduced effect of dopamine on reward sensitivity (PPC RS ON-OFF) [S^r=0.430, P=0.041], but this is not seen in PD patients without ICDs.

In PD patients without ICDs, increased apathy (LARS total) correlates with decreased mean overall pupil reward sensitivity (ON and OFF) [S^r=-0.400, P=0.043] as well as ON dopamine [S^r=-0.466, P=0.016]. These correlations are not found in PD patients with ICDs.

2. Subgroup analyses

2.1 <u>Current ICD vs prior ICD</u>

We investigated whether there were differences between these subgroups – current ICD (n=15) and past ICD (n=8). There were no significant differences in terms of UPPS-P or BISBAS scores, or subscales scores, or with respect to cognitive impairment (MOCA), depression (BDI) or apathy (LARS total). There was a significant difference between the 2 subgroups in LEDD, with current ICD cases having higher LEDD [unpaired t(21)=2.520, P=0.020].

Next, we performed a 3 Reward x 2 Drug x 2 Group repeated measures ANOVA on proportional pupil change (PPC). There was a significant main effect of Reward [F(2,42)=14.239, P<0.001] and being ON/OFF drug [F(1,21)=6.738, P=0.017], as we found in the main analysis. However, there was no significant 3-way interaction between Reward, Drug and Group, and no main effect of Group (unlike in the main analysis when we compared all PD+ICD to PD-no-ICD patients).

Importantly, there were no significant differences between these groups in terms of *proportional* pupil change reward sensitivity (autonomic reward sensitivity) ON dopamine, OFF dopamine, overall mean, or ON minus OFF (the effect of dopamine on reward sensitivity).

For our purposes the effect of dopamine on pupil reward sensitivity (PPC RS ON-OFF) is the most meaningful index to demonstrate that the primary group effect is not driven by the fact that some of the PD+ICD group have current ICDs and some only previously. Even in just the subgroup of PD+ICD with current ICDs (n=15), the findings from the original group analysis still hold, but with smaller effects due to the smaller sample size.

Importantly, PD+ICD patients with current ICDs had significantly lower LEDD levels than PD+ICD patients without current (active) ICD symptoms, but – despite this -– had no significant difference in proportional pupil change reward sensitivity, providing further evidence that behavioural impulsivity is not simply linearly related to dopamine dose.

We also note that despite not self-reporting ICD on QUIP, there may still be features of impulsivity in such patients, because there is significant underreporting. ICD is likely to be a manifestation of an underlying phenotype conferring propensity to develop an ICD.

2.2 <u>PD+ICD cases on only levodopa versus those also on agonists</u>

In order to assess whether there were differences in reward sensitivity between PD+ICD patients who were just on levodopa compared to those who were also taking agonists we performed a 3 Reward x 2 Drug x 2 Group repeated measure ANOVA on proportional pupil change. There was a significant main effect of Reward [$F(2,42)=19.540$, $P=<0.001$] and being ON/OFF drug [$F(1,21)=7.596$, $P=<0.05$], as we found in the main analysis, as well as a significant interaction between Reward and Group [$F(2,42)=4.088$, $P<0.05$] indicating differences in the way these two subgroups responded to rewarding stimuli. However, the crucial 3-way interaction between Reward, ON/OFF Drug and Group was not significant, and there was no main effect of Group.

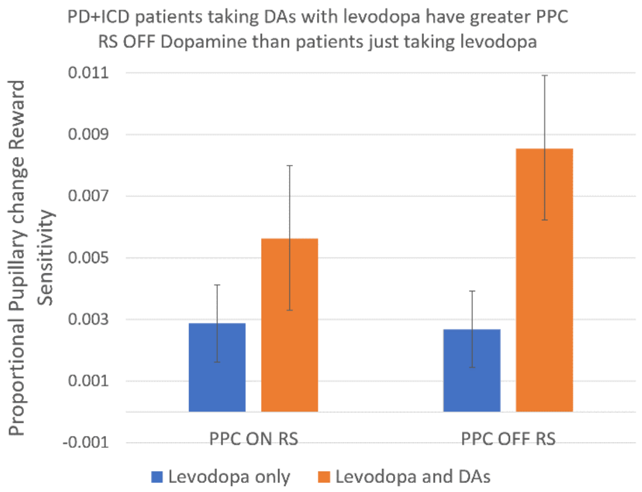

There was no significant difference between these two groups with respect to proportional pupil change reward sensitivity (autonomic reward sensitivity) ON dopamine. However, the PD+ICD patients currently on DA agonists had significantly greater pupil response to reward OFF dopamine [$F(1,21)=5.368$, $P=0.031$] – see figure above. In addition, they also had a significantly higher mean of ON and OFF dopamine pupillary response [$F(1,21)=5.814$, $P=0.025$], so it appears that being on DA agonists as well as levodopa is associated with increased overall reward sensitivity, driven by increased RS OFF dopamine. But, crucially, there was no significant difference between the two groups in the effect of dopamine on reward sensitivity (RS ON-OFF).

Is there an effect of dopamine agonist withdrawal on pupillary reward sensitivity?

The PD+ICD group also consists of some patients who have already undergone dopamine (DA) agonist withdrawal (i.e. with incomplete success). 21 PD+ICD patients had a history of taking DA agonists (ropinirole n=15, pramipexole n=5, rotigotine n=1). Of these 21 cases, 9 had withdrawn from DA agonists entirely, 1 had reduced DA agonist dose by half (Ropinirole 24mg to 12mg per day), approximately 6 years before testing. Of the 11 PD+ICD cases who were not on DA agonists, 9 had previously been on DA agonist, indicating that the drug was removed but this failed to resolve the ICD entirely. We managed to obtain the withdrawal dates for 5 of the 9 patients who had undergone DA withdrawal and this occurred a mean of 5.4 years before behavioural testing (range: 2.5 – 10 years). This information can be found in Supplementary Table (2).

Furthermore, 70% of PD+ICD patients presented with co-occurring apathy at the time of study. It has been previously reported that apathy and ICDs may co-occur, but we wanted to assess whether the physiological findings could relate to present apathy rather than prior or current ICDs. As mentioned above, 9 of the PD+ICD cases had experienced complete dopamine agonist withdrawal which is known to frequently cause apathy as part of the so-called “dopamine agonist withdrawal syndrome”. To look at this further we split the PD+ICD group into those who had experienced dopamine agonist withdrawal (n=9) and those who had not (n=14).

There was no significant increased apathy (LARS Total) in PD+ICD patients who had experienced dopamine agonist withdrawal (n=9) compared to those who had not (n=14), yet there was a significant difference in pupillary reward sensitivity between these subgroups OFF dopamine [unpaired t(17.1)=2.667, P=0.016] and mean overall pupil reward sensitivity [unpaired t(18)=2.783, P=0.012] indicating that PD+ICD patients who have undergone agonist withdrawal had reduced reward sensitivity than patients who have not. However, this reduction in reward sensitivity may not relate to agonist withdrawal but rather the fact that they are not currently taking agonists. We have shown above that PD+ICD patients currently taking agonists have greater reward sensitivity OFF, but not ON dopamine and that this may relate to the longer half-life of agonists compared to levodopa and also suggests that pupil reward sensitivity may relate more to impulsivity than apathy. There were no other differences between these subgroups in PD severity (UPDRS), cognitive impairment (MOCA), depression (BDI) apathy (LARS Total) or impulsivity (BISBAS/UPPS-P) except in the LARS subscale action initiation in which PD+ICD patients who have undergone agonist withdrawal actually had higher levels of action initiation (mean = -2.94) than patients who have not withdrawn from agonist use (mean = -2.11).

2.3 PD+ICD with Current ICDs (n = 15) vs PD-no-ICD (n = 26). RM (3x2x2) ANOVA on Proportional Pupil Change

We have already shown that there are no differences in pupil reward sensitivity between PD+ICD patients with current versus previous ICDs. Nevertheless we wanted to see whether our main reward sensitivity findings were preserved if we included only patients with current ICDs. We performed a 3 Reward x 2 Drug x 2 Group repeated measures ANOVA on proportional pupil change and found a significant main effect of Reward [F(2,78)=20.164, P=<0.001] and Drug [F(1,39)=10.123, P=0.003], as we found in the main analysis. Crucially, the 3-way interaction between Reward, Drug and Group [F(2,78)=5.086, P=0.008] was still significant, as was the main between subjects effect of group [F(1,39) = 12.989, P<0.001] that we found in the main analysis between PD+ICD and PD-no-ICD patients.

Therefore, the main effects from our primary analysis on pupillary reward sensitivity are maintained when analysing only the PD+ICD patients with current ICDs, despite a considerably smaller sample size.

2.4 Could the overall PD+ICD result of greater reward sensitivity in the OFF condition compared to PD-no-ICD all be attributed to the PD+ICD cases on DA agonists?

Considering that PD+ICD patents on agonists have greater pupil reward sensitivity OFF dopamine than PD+ICD patients on only Levodopa, we were wondering to what extent our main reward sensitivity group effect was driven by these patients. We performed a 3 Reward x 2 Drug x 2 Group repeated measure ANOVA on proportional pupil change and excluded the patients currently taking agonists from the PD+ICD group: (PD+ICD on only levodopa (n=11) vs PD-no-ICD (n=26).

This analysis revealed a significant main effect of Reward [F(2,70)=10.914, P<0.001] and Drug [F(1,35)=11.994, P=0.001], as we found in the main analysis. Importantly, the 3-way interaction between Reward, Drug and Group [F(2,88)=3.845, P=0.026] was still significant, as was the main between subjects effect of Group [F(1,35)=5.607, P<0.024].

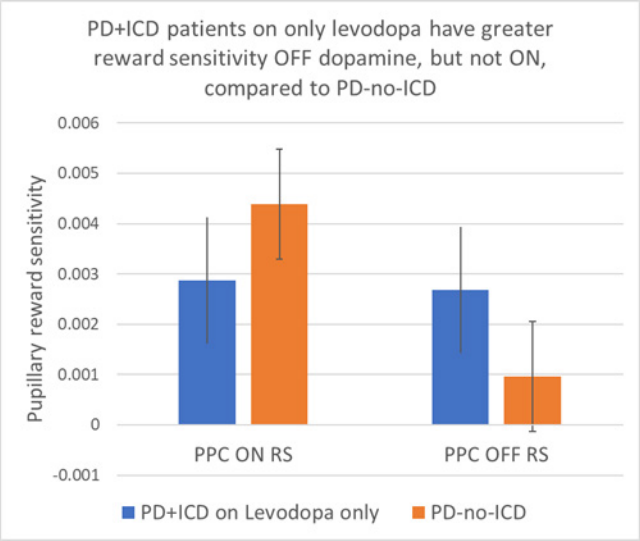

Even when excluding all PD+ICD patients who were currently taking dopamine agonists at the time of testing the main findings reported in the text are preserved, despite a significantly smaller sample size. This is particularly interesting considering that PD+ICD patients on agonists have even greater reward sensitivity (OFF dopamine) than the PD+ICD patients only taking Levodopa.

Nevertheless, the key point is that excluding the patients currently taking agonists did not change the main effects. Although these patients still had increased reward sensitivity the main effects are not entirely driven by the patients taking agonists. In other words, the increased pupil reward sensitivity seen in these PD patients is not all attributable to dopamine agonist use, but it can be exacerbated by it.

2.5 PD-no-ICD cases on only levodopa versus those also on agonists

In the PD-no-ICD group, 11 patients were taking DAs at the time of behavioural testing and 15 were only taking levodopa. We found no evidence that the DA dose had been reduced over time in any of these patients. Of the 15 patients only taking levodopa, for 11 we found no evidence that they had previously taken DAs, but for 4 of these patients we could not access their clinical notes.

We assessed which of the PD-no-ICD patients went on to develop ICD in the subsequent 5 years and whether this was influenced by the type of medication they were taking. Of the 12 patients taking dopamine agonists, 8 (73%) developed ICD in the 5 years since they performed our oculomotor task. Of the 13 patients who have only ever taken levodopa medication 6 (46%) went on to develop ICD.

We also performed a 3 Reward x 2 Drug ON/OFF x 2 Group repeated measures ANOVA on proportional pupil change (PPC) with two PD-no-ICD subgroups split by medication type: Only Levodopa vs Levodopa and dopamine agonists. We found a significant main effect of Reward [F(2,44) = 11.304, P<0.001] and Drug condition (ON/OFF) [F(1,22) = 4.892, P=0.038] but no main between subjects effect of Group. Furthermore there was a significant interaction between Drug and Reward [F(2,44)=6.026, P=0.005] and a 3-way interaction between Drug, Reward and Group [F2,44)=3.581, P=0.036].

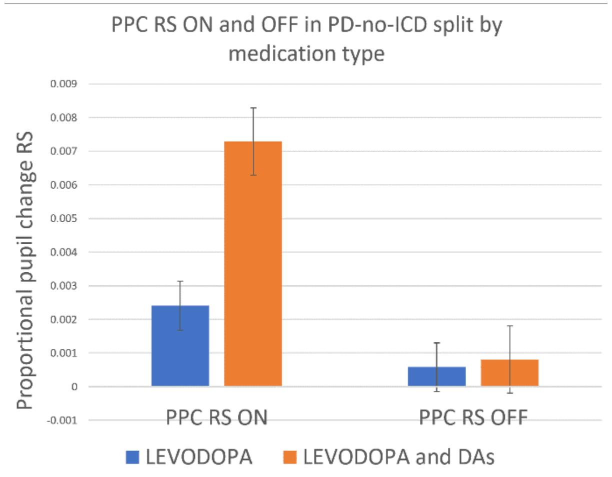

The 3-way Reward x ON/OFF Drug x Group interaction in this analysis was significant, showing that in these PD-no-ICD cases, the pupillary response to reward is greater for individuals on dopamine agonists plus levodopa, compared to those on levodopa alone in the ON medication condition, but not OFF (see figure). Note, however, that OFF medications the pupillary response for these PD-no-ICD cases remains very low – significantly lower than the PD+ICD cases who have heightened reward sensitivity OFF dopamine (compare to analogous figure for PD+ICD cases shown earlier).

Furthermore, there is a significant difference in the effect of overnight withdrawal on pupil reward sensitivity between these two subgroups, with PD-no-ICD patients on agonists having a greater increase ON dopamine than OFF, compared to PD-no-ICD patients only taking levodopa.

There was no significant difference between the two subgroups in terms of impulsive traits (UPPS-P, BISBAS subscales), apathy (LARS), depression (BDI) or overall LEDD.
