# Supplementary material for: Dopamine and reward hypersensitivity in Parkinson’s disease with impulse control disorder
Source: Brain. 2020 Aug 6;143(8):2502–18. doi: 10.1093/brain/awaa198 (PMC7447523; doi:10.1093/brain/awaa198)
Supplement: awaa198_Supplementary_Data [file awaa198_supplementary_data.zip › awaa198-suppl_data/brain-2019-01617-File011.pdf]

1.13. a: **Scatter plot showing pupillary reward sensitivity for each subject ON and OFF dopamine.**

This scatter plot shows the relationship between reward sensitivity ON (X-axis) and OFF (Y-axis) dopamine in PD+ICD (red) and PD-no-ICD (blue). Subjects nearer the top right corner have greater overall pupillary reward sensitivity and subjects nearer the bottom left corner have lower overall reward sensitivity. Subjects above green line have greater reward sensitivity OFF dopamine, whereas subjects under the green line have greater reward sensitivity ON dopamine. 60% (14/23) of PD+ICD patients have greater pupil reward sensitivity OFF dopamine compared to only 19% (5/26) of PD-no-ICD patients.

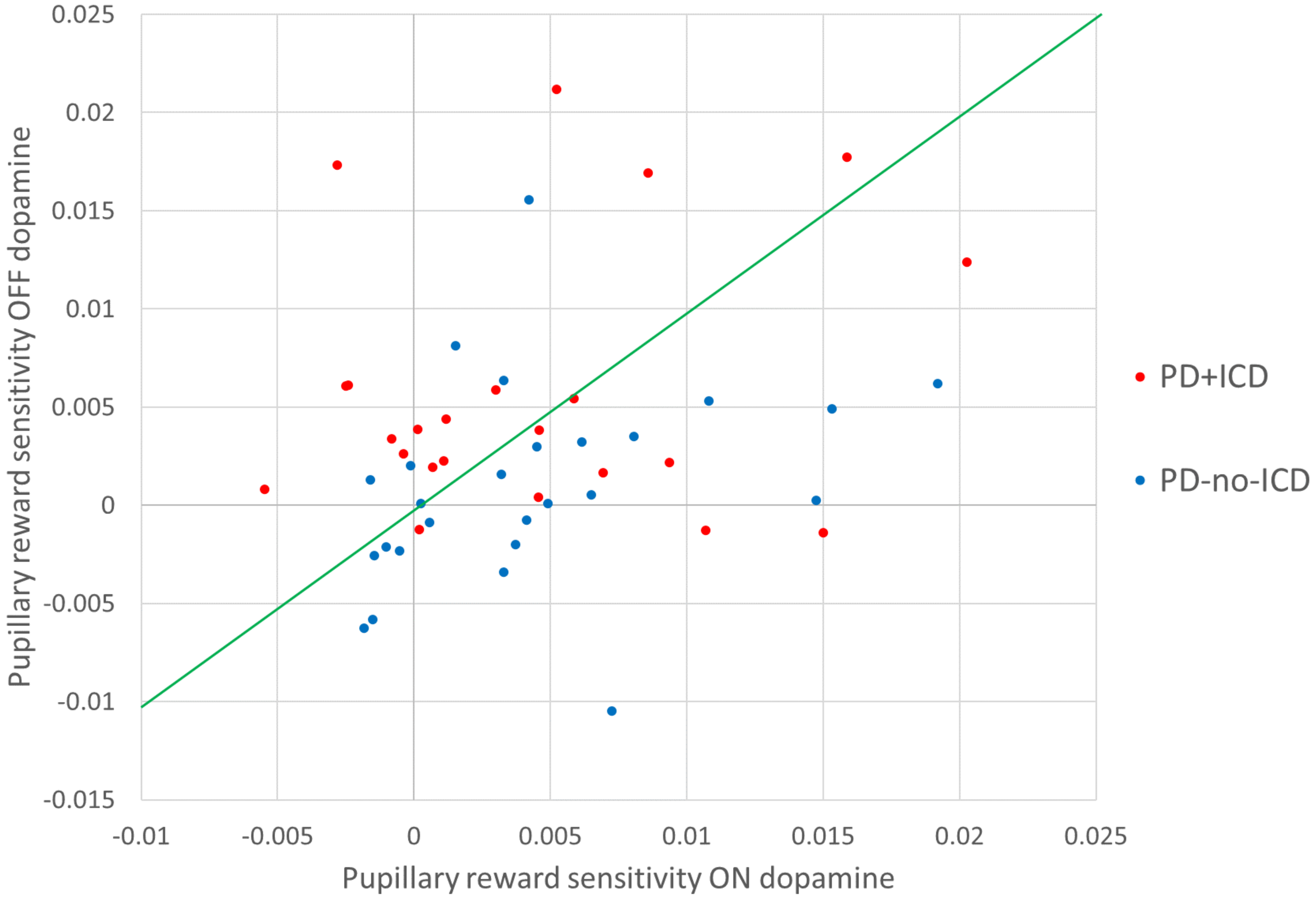

0.025
0.02
0.015
0.01
0.005
0
-0.005
-0.01
-0.015
-0.01
-0.005
0
0.005
0.01
0.015
0.02
0.025
Pupillary reward sensitivity OFF dopamine
Pupillary reward sensitivity ON dopamine
PD+ICD
PD-no-ICD

1.13. b: Histogram showing pupillary reward sensitivity with paired datapoints for each subject ON and OFF dopamine.

Mean proportional pupil reward sensitivity (mean pupillary change for 50p condition minus 0p condition) for PD+ICD (red) and PD-no-ICD (blue) ON and OFF dopaminergic medication with paired datapoints for each subject ON and OFF dopamine. In PD-no-ICD withdrawing dopamine significantly reduced pupillary reward sensitivity but this was not observed in PD+ICD.

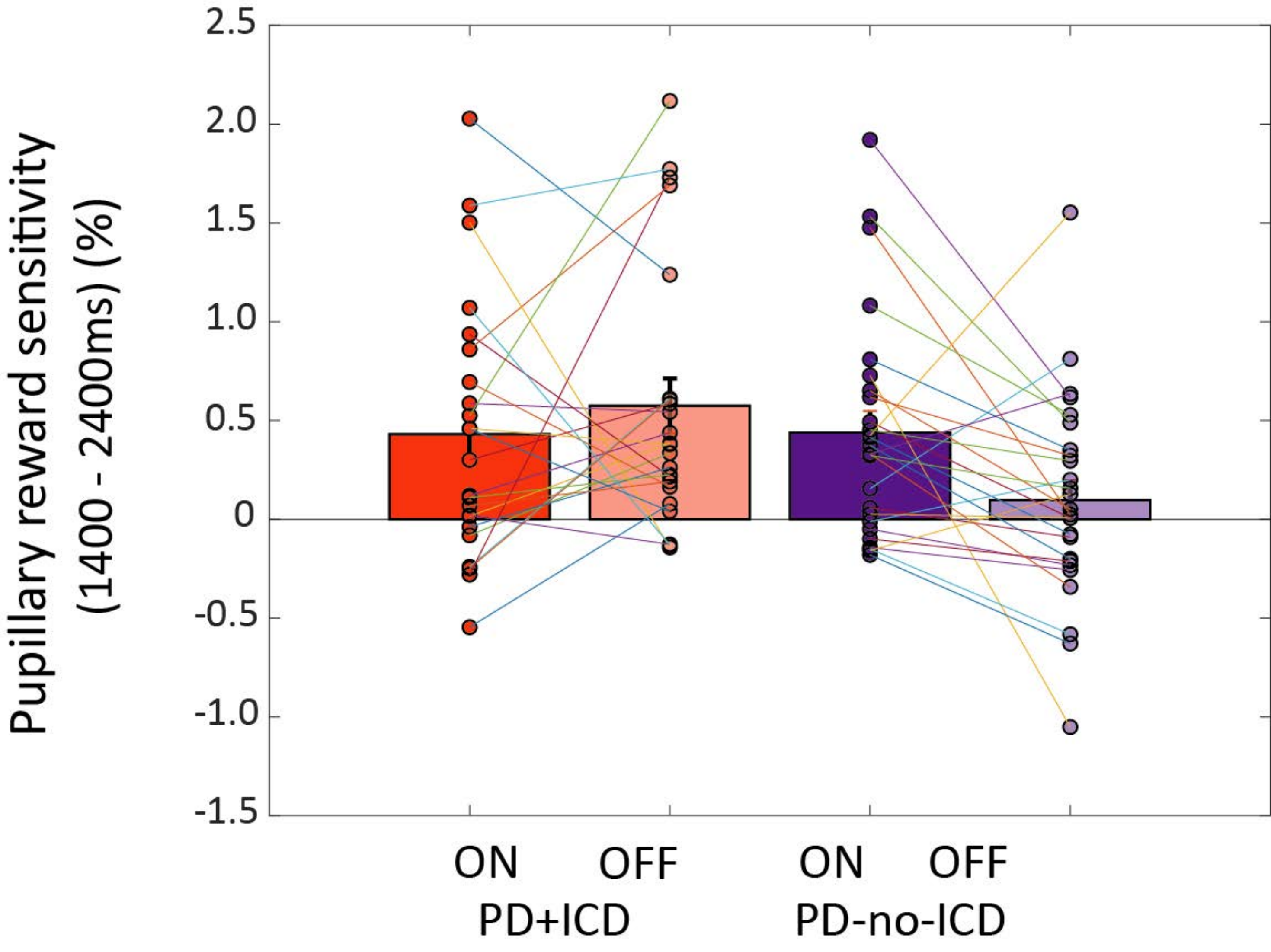

Pupillary reward sensitivity
(1400 - 2400ms) (%)
2.5
2.0
1.5
1.0
0.5
0
-0.5
-1.0
-1.5
ON
OFF
ON
OFF
PD+ICD
PD-no-ICD

1.13. c: Histogram showing mean peak saccadic residual velocity reward sensitivity with paired datapoints for each subject ON and OFF dopamine.

Mean peak *residual* velocity reward sensitivity (mean peak residual velocity for 50p condition minus 0p condition) for PD+ICD ON (red) and OFF (light red), and for PD-no-ICD ON (blue) and OFF (light blue) with paired datapoints for each subject ON and OFF dopamine. No significant effect of dopamine on residual velocity reward sensitivity was observed in PD+ICD, whereas in PD-no-ICD residual velocity reward sensitivity was significantly greater ON dopamine, compared to OFF. However, PD+ICD did not exhibit greater overall residual velocity or greater reward sensitivity than PD-no-ICD. Furthermore, there was no significant difference between PD+ICD and PD-no-ICD ON or OFF medication although there is a trend towards PD+ICD having greater RS OFF dopamine [P=0.066].

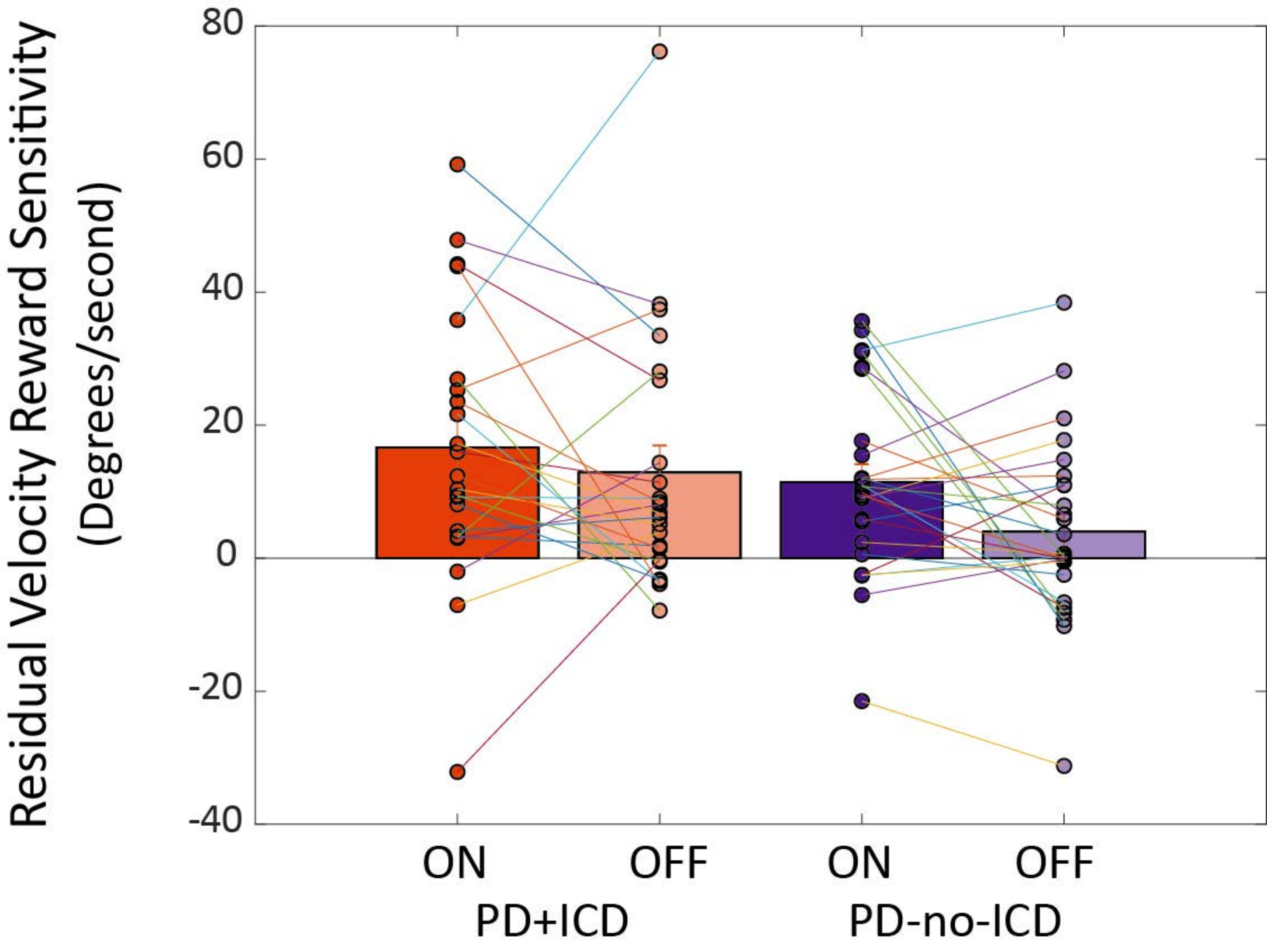
Residual Velocity Reward Sensitivity
(Degrees/second)
80
60
40
20
0
-20
-40
ON
OFF
ON
OFF
PD+ICD
PD-no-ICD

Figure 1.13. d: Histogram showing mean standard deviation of saccadic amplitude reward sensitivity with paired datapoints for each subject ON and OFF dopamine

Mean standard deviation of saccadic amplitude reward sensitivity in PD+ICD ON (red) and OFF (light red) and PD-no-ICD ON (blue) and OFF (light blue) dopaminergic medication with paired datapoints for each subject ON and OFF dopamine. No difference was found between the reward sensitivity ON and OFF dopamine in PD+ICD whereas in PD-no-ICD there was significantly reduced variability of saccadic amplitude reward sensitivity (error) ON dopamine compared to OFF.

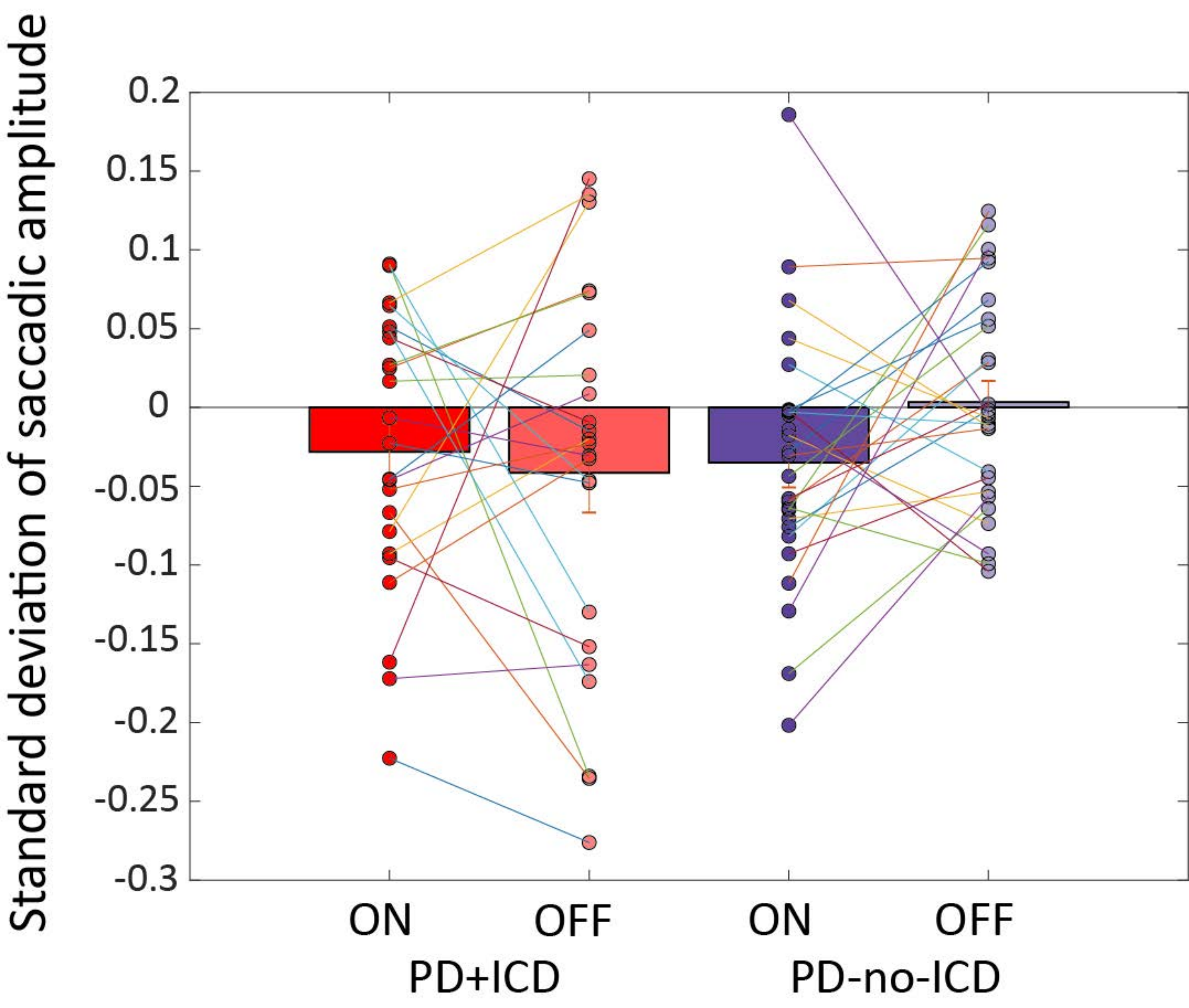

Standard deviation of saccadic amplitude
0.2
0.15
0.1
0.05
0
-0.05
-0.1
-0.15
-0.2
-0.25
-0.3
ON
OFF
ON
OFF
PD+ICD
PD-no-ICD
